# Design and Antioxidant Properties of Bifunctional 2*H*-Imidazole-Derived Phenolic Compounds—A New Family of Effective Inhibitors for Oxidative Stress-Associated Destructive Processes

**DOI:** 10.3390/molecules26216534

**Published:** 2021-10-29

**Authors:** Elena L. Gerasimova, Elena R. Gazizullina, Maria V. Borisova, Dinara I. Igdisanova, Egor A. Nikiforov, Timofey D. Moseev, Mikhail V. Varaksin, Oleg N. Chupakhin, Valery N. Charushin, Alla V. Ivanova

**Affiliations:** 1 Institute of Chemical Engineering, Ural Federal University, 620002 Ekaterinburg, Russia; e.l.gerasimova@urfu.ru (E.L.G.); e.r.gazizullina@urfu.ru (E.R.G.); mborisova97@icloud.com (M.V.B.); igdisanova.2011@mail.ru (D.I.I.); e.a.nikiforov@urfu.ru (E.A.N.); timofey.moseev@urfu.ru (T.D.M.); m.v.varaksin@urfu.ru (M.V.V.); chupakhin@ios.uran.ru (O.N.C.); valery-charushin-562@yandex.ru (V.N.C.); 2 Postovsky Institute of Organic Synthesis, Ural Branch of the Russian Academy of Sciences, 620990 Ekaterinburg, Russia

**Keywords:** 2*H*-imidazole, polyphenols, antioxidant capacity, antiradical capacity

## Abstract

The synthesis of inhibitors for oxidative stress-associated destructive processes based on 2*H*-imidazole-derived phenolic compounds affording the bifunctional 2*H*-imidazole-derived phenolic compounds in good-to-excellent yields was reported. In particular, a series of bifunctional organic molecules of the 5-aryl-2*H*-imidazole family of various architectures bearing both electron-donating and electron-withdrawing substituents in the aryl fragment along with the different arrangements of the hydroxy groups in the polyphenol moiety, namely derivatives of phloroglucinol, pyrogallol, hydroxyquinol, including previously unknown water-soluble molecules, were studied. The structural and antioxidant properties of these bifunctional 5-aryl-2*H*-imidazoles were comprehensively studied. The redox transformations of the synthesized compounds were carried out. The integrated approach based on single and mixed mechanisms of antioxidant action, namely the AOC, ARC, Folin, and DPPH assays, were applied to estimate antioxidant activities. The relationship “structure-antioxidant properties” was established for each of the antioxidant action mechanisms. The conjugation effect was shown to result in a decrease in the mobility of the hydrogen atom, thus complicating the process of electron transfer in nearly all cases. On the contrary, the conjugation in imidazolyl substituted phloroglucinols was found to enhance their activity through the hydrogen transfer mechanism. Imidazole-derived polyphenolic compounds bearing the most electron-withdrawing functionality, namely the nitro group, were established to possess the higher values for both antioxidant and antiradical capacities. It was demonstrated that in the case of phloroglucinol derivatives, the conjugation effect resulted in a significant increase in the antiradical capacity (ARC) for a whole family of the considered 2*H*-imidazole-derived phenolic compounds in comparison with the corresponding unsubstituted phenols. Particularly, conjugation of the polyphenolic subunit with 2,2-dimethyl-5-(4-nitrophenyl)-2*H*-imidazol-4-yl fragment was shown to increase ARC from 2.26 to 5.16 (10^4^ mol-eq/L). This means that the considered family of compounds is capable of exhibiting an antioxidant activity via transferring a hydrogen atom, exceeding the activity of known natural polyphenolic compounds.

## 1. Introduction

Free radical processes and the degree of their intensification are known to be significant pathogenetic factors in the progress of many diseases [1,2]. On the one hand, the specificity and intensity of the oxidative stress processes could differ in various tissues depending on their structural organization, characteristic features of biochemical processes and functional activities. On the other hand, the state of oxidative stress often affects the entire body. Additionally, the excessive formation of reactive oxygen metabolites (ROS) on cellular structures in a wide range of pathological conditions, such as pathologies of the cardiovascular and nervous systems, endocrine diseases, in particular diabetes mellitus, oncology, and many others, currently proves to play a crucial role in the destructive processes [3,4,5,6,7].

Oxidative stress affects almost all structures in the body, including DNA, proteins, and membrane lipids; it results in disorders of homeostasis at both cellular and tissue levels, namely the imbalance between pro- and anti-inflammatory cytokines, the endothelial dysfunction, the vegetative and mediator dysfunction of cells, mitochondrial dysfunction, etc. [3,4,5,6,7,8,9]. In this regard, antioxidants are widely used for the effective pharmacological control of pathological cellular responses underlying the oxidative stress. In addition, the combination of basic drugs for therapy and drugs with antioxidant actions has recently been shown to enhance the positive effect in the therapy of a wide range of cardiovascular, endocrine, and oncological diseases [10,11,12,13,14].

One of the current trends in the development of modern pharmacology is known to be the target design of drugs with a combined mechanism of action. Numerous studies have demonstrated that the use of the action mechanism of drugs, i.e., application of bioactive compounds involved in several biochemical processes enables the effectiveness of the therapy of various diseases to be significantly increased. This effect is generally achieved by using the strategy of drug combinations [13,14,15,16,17,18,19]. At the same time, one of the promising methods for the further development of this direction is to apply bi- and polyfunctional organic molecules of the azaheterocyclic series with the multitargeting mechanisms of action for the treatment of diseases caused by oxidative stress. Despite the fact that conjugation can often lead to a decrease in the activity of both fragments of a hybrid molecule, some authors have succeeded in synthesizing compounds where the activity of the conjugated molecule exceeded the activity of the individual compound [20,21].

**Figure 1:**
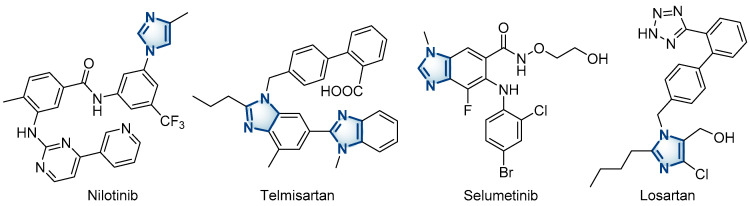
Active pharmaceutical ingredients (APIs) and approved drugs based on the imidazole structural motif.

The imidazole scaffold is currently considered as one of the most attractive structural units in bioactive molecules of both natural and synthetic origin. Notably, imidazole derivatives are widely exploited in the design of active pharmaceutical ingredients (APIs) and drugs based on them, effective against diseases of various etiologies [22,23,24] (Figure 1). For instance, Nilotinib is a kinase inhibitor used for the chronic phase treatment of Chronic Myeloid Leukemia (CML) [25]; Telmisartan is an angiotensin receptor blocker (ARB) used to treat hypertension, diabetic nephropathy, and congestive heart failure [26]; Selumetinib is a mitogen-activated protein kinase (MEK) 1/2 inhibitor used in pediatric patients to treat neurofibromatosis type 1 (NF1) accompanied by symptomatic, inoperable plexiform neurofibromas (PN) [27]; Losartan is an angiotensin receptor blocker used to treat hypertension and diabetic nephropathy, and is used to reduce the risk of stroke [28].

Meanwhile, functional derivatives of polyphenols deserve also a special attention in pharmaceutical chemistry due to a variety of biological activities, such as antitumor, antiviral, and anti-inflammatory ones [29,30,31]. In addition, the antioxidant/antiradical properties of polyphenols underlie the action mechanism of drugs used for the prevention and therapy of cancer, coronary arteriosclerosis, and degradation aging processes caused by the phenomenon of the oxidative stress [32,33], which are able to be efficiently inhibited following the various mechanisms of the antioxidant action [14,15,16,17,18].

**Figure 2:**
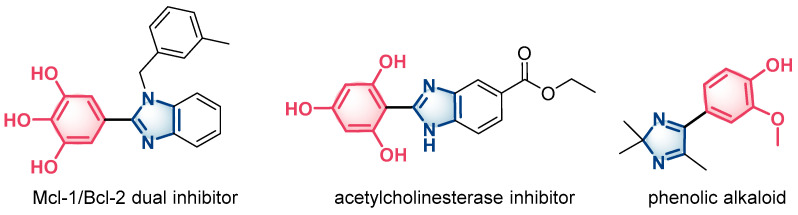
Examples of azaheterocyclic phenolic compounds in the design of candidates for active pharmaceutical ingredients (APIs).

In this regard, small organic molecules of a certain chemical architecture, in which the phenolic scaffold is attached directly to the azaheterocyclic backbone, can be considered as promising drug candidates for the treatment of socially significant diseases, such as neurodegenerative, cardiovascular, oncological diseases, etc. In particular, one can observe a few examples of 2*H*-imidazole-derived phenolic compounds of practical interest such as a Mcl-1/Bcl-2 dual inhibitor that effectively induces apoptosis in a dose-dependent, mechanism-based manner in multiple cancer cell lines, an acetylcholinesterase inhibitor (AChE), and a phenolic alkaloid derived from the plant *Dracocephalum heterophyllum* (Figure 2) [34,35,36].

It should also be noted that antioxidants are quite multifaceted in the progress of many diseases associated with the phenomenon of oxidative stress [1,2,3,4,5,6]. Therefore, the issue of their practical use should be regarded in view of their action mechanisms. There are several conventional ways to intensify the production of active oxygen metabolites, and the participation of antioxidants in them [3,4,5,6,7,8,9]. The first one involves the mitochondrial dysfunction associated processes. In this case, one should focus on the therapy features with inhibitors of radical reaction, i.e., with chemical agents acting in accordance with the hydrogen atom transfer (HAT) mechanism. The second way is associated with the concept of the cell redox potential. The accumulation of under-oxidized destruction products of proteins and lipids in the cell leads to the pronounced changes in the redox potential of the corresponding cell membranes. The latter disrupts the entry processes of substances into the cell, which are necessary for its normal functioning, and to realize the processes of excretion of decay products. This process is directly related to the action of antioxidants through the electron transfer (ET) mechanism. The third way is that tissues containing iron ions can react with hydrogen peroxide, toxic hydroxyl radicals being produced by the Fenton reaction. Acting on the destructive reactions’ intermediates, the chelation of iron and copper ions can be another defense option.

Thus, in order to access antioxidant actions for the designed organic molecules of interest in the prevention and treatment of socially significant diseases associated with the oxidative stress, the complex approach providing in-depth analysis of antioxidant properties from the standpoint of various mechanisms of action needs to be exploited [37,38,39,40,41]. This work is devoted to the study of the antioxidant properties for bifunctional organic compounds of the 5-aryl-2*H*-imidazoles series modified with polyphenol fragments (phloroglucinol, pyrogallol, hydroxyquinol) and also revealing the correlations “structure-antioxidant activity” to find out lead compounds in the design of drug candidates. Herein, the integrated approach that allows research from the standpoint of various mechanisms of antioxidant/radical-inhibiting action [40,41,42,43] was first used to estimate antioxidant properties of the considered imidazole-derived polyphenolic molecular systems.

## 2. Results

**Figure 3:**
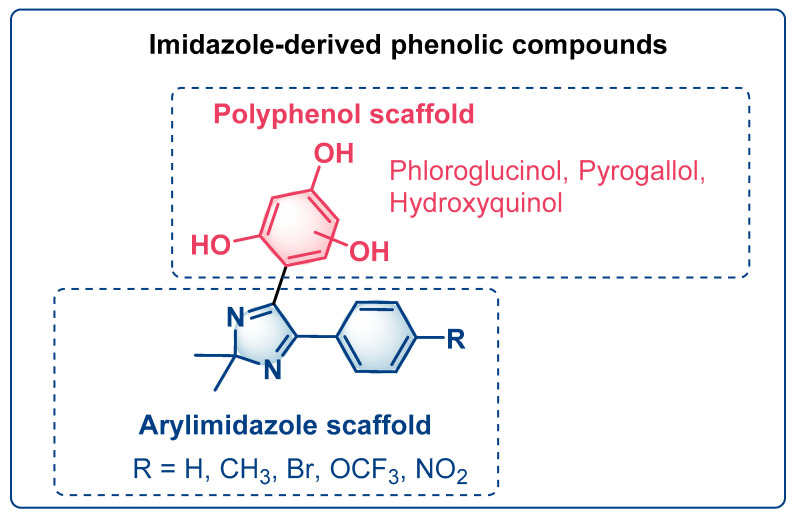
Imidazole-derived phenolic compounds.

In this work, we studied functional derivatives of the arylimidazole scaffold modified with polyphenol residues (Figure 3), such as phloroglucinol, pyrogallol, hydroxyquinol, i.e., small molecules, in which the biogenic azaheterocyclic fragment is responsible for selective binding with bioreceptors, while the polyphenolic fragment provides the antioxidant/radical-binding effects.

**Scheme 1:**
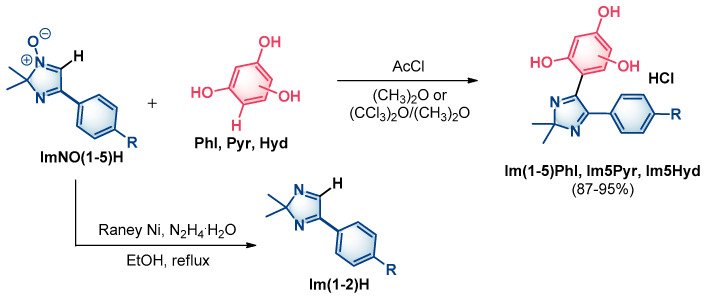
Synthesis of 2*H*-imidazole-derived phenolic compounds **Im(1–5)Phl**, **Im5Pyr**, **Im5Hyd** and 2*H*-imidazoles **Im(1–2)H**.

The design and synthesis of water-soluble bifunctional molecular systems, bearing both 2*H*-imidazole and polyphenolic building blocks, for the study of their antioxidant activity as challenging drug candidates with a combined mechanism of action, was carried out in accordance with the established synthetic methodology [44]. The synthetic strategy is based on the pot and atom economy methodology of C-H functionalization of non-aromatic azaheterocyclic substrates that was realized as reactions of nucleophilic substitution of hydrogen (S_N_^H^) in 2*H*-imidazole 1-oxide [45,46,47,48,49]. The targeted synthesis of arylimidazoles bearing functional groups in the *para*-position of the aryl fragment was carried out to assess at the next step the effects of various substituents on antiradical properties of the investigated bifunctional compounds. The reaction of 2*H*-imidazole-*N*-oxides **ImNO(1–5)H** with polyphenols **Phl**, **Pyr**, **Hyd** proved to proceed in the presence of AcCl, thus leading to a series of previously unknown *2H*-imidazole-derived phenolic compounds, such as **Im(1–5)Phl**, **Im5Pyr**, **Im5Hyd** in good-to-excellent yields. It should be noted that these 7 water-soluble bifunctional molecular systems were intentionally designed in this work and isolated in 87–95% yields (Scheme 1). In addition, we managed to prepare the reduced forms of some 2*H*-imidazole-*N*-oxides **Im(1–2)H** for the first time by using hydrazine hydrate in the presence of an activated Raney-nickel catalyst [50] to evaluate the contribution of the arylimidazole moiety to the overall antiradical properties of bifunctional molecular systems. The structures of 2*H*-imidazoles, polyphenols, and 2*H*-imidazole-derived phenolic compounds are shown in Table 1. All the synthesized compounds were fully characterized by the data of ^1^H, ^13^C-NMR and IR-spectroscopy, mass-spectrometry, and elemental analysis (Supporting Information contains copies of spectra for novel compounds).

### 2.1. Study of Electrochemical Behavior of Synthesized Compounds

Since the AO action of organic molecules is closely related with their ability to be oxidized, the redox properties of the synthesized compounds were evaluated at the first step of the study. One of the universal approaches to assess the redox properties of organic compounds is a method of cyclic voltammetry (CV). The presence of oxidation signals on the anodic branch of the CVA in the negative or near positive range of potentials could indicate the ability of compounds to facilitate the oxidation processes sufficiently.

**Figure 4:**
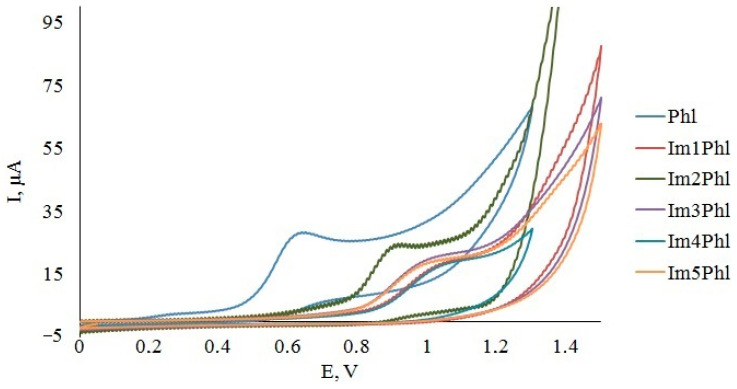
Cyclic voltammograms of phloroglucinol and imidazolylphloroglucinol recorded on the glassy carbon electrode (C = 1 mmol/dm^3^; background: 0.1 mol/dm^3^ KCl; scan rate 0.1 V/s).

**Figure 5:**
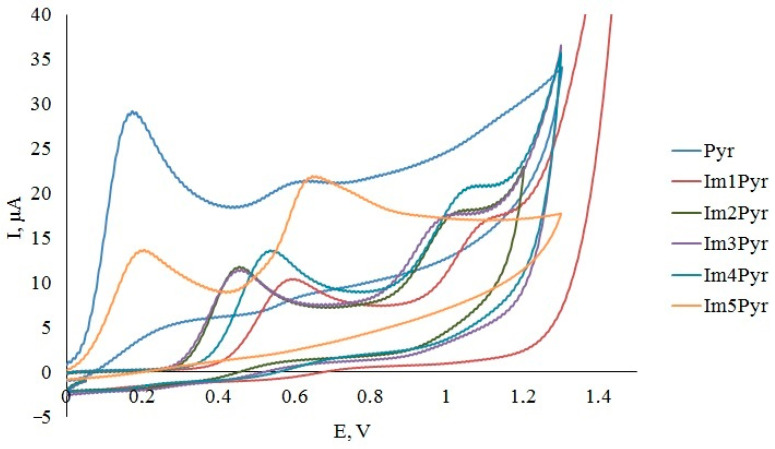
Cyclic voltammograms of pyrogallol and imidazolylpyrogallols recorded on the glassy carbon electrode (C = 1 mmol/dm^3^; background: 0.1 mol/dm^3^ KCl; scan rate 0.1 V/s).

**Figure 6:**
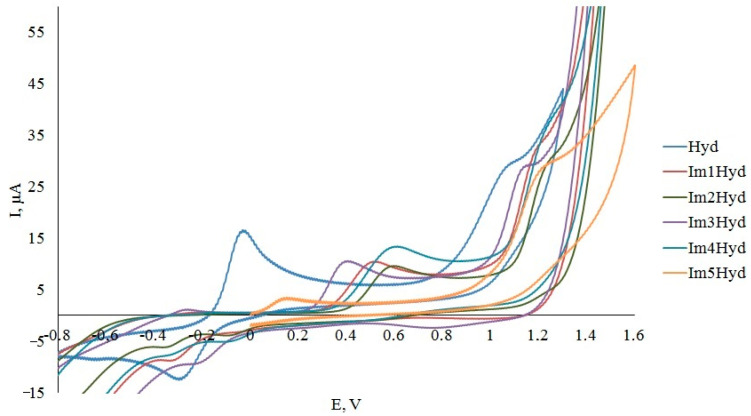
Cyclic voltammograms of hydroxyquinol and imidazolylhydroxyquinols recorded on the glassy carbon electrode (C = 1 mmol/dm^3^; background: 0.1 mol/dm^3^ KCl; scan rate 0.1 V/s).

It was established that the initial imidazoles showed no electrochemical activity. Figure 4, Figure 5 and Figure 6 demonstrate cyclic voltamperograms (CV) of imidazolylpolyphenols in comparison with the initial phenols.

Oxidation potentials of phenols and imidazolylpolyphenols and values of the current for oxidation peaks are presented in Table 2.

According to CVs and the data presented in Table 2, one can see that the conjugation of imidazole and polyphenolic fragments for all synthesized imidazolylpolyphenols shifts the anodic peak (the first anodic peak for imidazolylpyrogallols and imidazolylhydroxyquinols) to a more positive region.

It should also be noted that both pyrogallol and oxyhydroquinone are characterized by the presence of two anodic peaks. The position of the first oxidation peak is in the range of 0.14–0.57 V, the latter seems to indicate facilitation of electron transfer, and is typical for antioxidant compounds that implement the electron transfer mechanism as well. In this case, the conjugation of the imidazole and pyrogallol fragments does not cause a shift to the positive region, and, in some cases, shifts the potential of the first oxidation peak to the region of more negative values. Notably, the structurally similar compounds are also characterized by antioxidant properties realized through the hydrogen atom transfer mechanism, which is in an aqueous medium, combined with the electron transfer mechanism [38,39,40,41].

Phloroglucinol and its derivatives were found to be characterized by the presence of an oxidation peak in the rather positive range of 0.98–1.02 V, which indicates a hindered electron transfer process. Thus, it can be assumed that phloroglucinol, as well as imidazolylphloroglucinols seem to be involved not only in the electron transfer process; however, these molecules are capable of exhibiting activity through the chelation and the hydrogen atom transfer mechanisms [51,52,53].

### 2.2. Study of Antioxidant Properties of Polyphenols and Aynthesized Compounds

As described earlier, the integrated approach must be used to gain the most complete and reliable information on the antioxidant properties of the synthesized compounds [38,39]. Since there is a need for antioxidants, acting by various mechanisms, in the treatment of diseases associated with oxidative stress [3,4,5,6,7,8,9], it is necessary to evaluate antioxidant properties from the standpoint of their mechanisms of action. In this regard, polyphenolic compounds are capable of reacting with oxygen metabolites by various mechanisms [27,28,29,30,31] listed below:

- Electron-transfer (ET);
- Hydrogen atom transfer (HAT);
- Metal chelating mechanism.

In this work, four different approaches based on the model reactions implementing various mechanisms of antioxidant action, as well as their combinations were applied:

- Determination of antioxidant capacity (TAC): ET-mechanism + metal chelating mechanism [40,41,54,55,56];
- Determination of antiradical capacity (ARC): HAT-mechanism [40,56,57];
- Folin assay: ET-mechanism [43];
- DPPH assay: HAT-mechanism + ET-mechanism [42].

The integrated approach, based on the use of both conventional methods and well-founded approaches, will allow obtaining the most complete and reliable information on the antioxidant properties for a number of small organic molecules [40,41].

Table 3 shows the results of experiments performed for evaluation of antioxidant properties. As expected, antioxidant activity for the initial imidazoles by the presented methods was not displayed.

Since the studied compounds appear to be rather complex conjugated structures, and research methods are based on different approaches and mechanisms of antioxidant action, the unambiguous correlations were not established; nevertheless, a number of trends were identified.

As follows from the data presented in Table 3, in most cases (except the ARC value for the series of imidazolylphloroglucinols **Im1Phl**, **Im2Phl**, **Im5Phl**, **Im4Pyr**, and the AOC value for **Im5Hyd**) the conjugation of polyphenol and phenylimidazole fragments leads to a decrease of all values relative to the initial polyphenols. Similar regularities were reported earlier in the study of other bifunctional compounds [41].

According to the Folin assay, which is based on the electron transfer mechanism, this tendency was shown to be typical for all pyrogallol derivatives; however, the conjugation for oxyhydroquinone derivatives did not significantly change the results of the Folin assay (ET-mechanism). No activity due to the electron transfer mechanism was observed for phloroglucinol, and the incorporation of the imidazole moiety did not result in its appearance. This pattern is consistent with the data obtained by the cyclic voltammetry method. Low values and their insignificant changes in the series of compounds could be due to the fact that the interaction with the oxidizing agent in this method is carried out under acidic conditions [38,43]. It is known that the electron-donor ability of polyphenolic compounds in an acidic medium is rather low because of the formation of associates [58]. This may also be a reason for the absence of a noticeable effect of conjugation with the imidazole moiety on the results of the Folin assay. This fact does confirm the inadequacy of using one approach.

The antioxidant capacity is known to be an integral value reflecting the contribution of antioxidant activity through both the electron transfer and the chelation mechanisms [40,41,54,55,56]. The considered polyphenolic compounds were reported to exhibit a tendency to form complexes with iron ions [51,53]. Generally, conjugation with the polyphenol moiety was shown to decrease the value of the antioxidant capacity, while introduction of substituents (Br, F_3_CO) into the phenylimidazole fragment results in the AOC values to be decreased relative to the corresponding imidazole-derived polyphenols with imidazole **Im1** and **Im2** scaffolds. It was also found that the incorporation of NO_2_ group into the imidazolol fragment does not significantly affect the AOC values of imidazolylphloroglucinols, which is likely due to the chelation effect, but leads to a significant increase in the AOC values for imidazolylpyrogallols and imidazolylhydroxyquinols. In this case, the AOC is associated with both the chelation effect and the electron transfer process. This observation can be associated with the pronounced electronegative properties of the nitrobenzene functionality that shifts the electron density, thus facilitating the phenolic fragment oxidation. This feature was also confirmed by the CV data (Table 2); the potentials of the first oxidation peaks proved to be shifted to a more negative region.

As expected, an inverse correlation between the position of the oxidation peak (the first peak for pyrogallol and oxyhydroquinone derivatives) and the AOC value was observed, namely for pyrogallol derivatives (R = 0.89, *n* = 5, r_crit_ = 0.75) and oxyhydroquinone (R = 0.87, *n* = 5, r_crit_ = 0.75) (Figures S20 and S21 in Supplementary Materials). In particular, the more negative location is demonstrated by the anodic peak of imidazolylpolyphenols, the more facilitated the process of electron transfer from the polyphenol fragment, and the higher the AOC values are registered.

The DPPH assay results cannot be interpreted unambiguously because of two plausible factors, such as a mixed mechanism for interaction of stable radicals in the DPPH assay and antioxidants, and a water–ethanol medium used for the reaction [39,42], which is quite far from physiological conditions. While the physiological conditions of the study of antioxidant properties are the most appropriate, they are implemented in potentiometric approaches to determine the antioxidant and antiradical capacity.

The value of the antiradical capacity was found to reflect the antioxidant properties based on the hydrogen atom transfer mechanism [40,41,56,57]. A number of valuable patterns were also observed herein. In particular, the conjugation of the polyphenolic and phenylimidazole moieties for pyrogallol derivatives did not affect the value of the antiradical capacity, as oxyhydroquinone derivatives lead to its decrease. However, the opposite patterns were obtained for phloroglucinol derivatives, where incorporation of the phenylimidazole fragment enhances the antiradical capacity of **Im1Phl**, **Im2Phl**, and **Im5Phl** compounds. This may be due to the formation of stable radicals of imidazolylpolyphenols, when they interact with a model oxidant of the radical nature. Remarkably, this effect is a more characteristic one for the compounds bearing the phloroglucinol fragment relative to pyrogallol and oxyhydroquinone derivatives. In this case, the introduction of a strong electronegative substituent into the phenylimidazole fragment was found to result in a greater stabilization of the forming phenyl radical. This process contributes to a shift of the reaction equilibrium with a model oxidant towards the formation of products and enables one to obtain rather high values of the antiradical capacity. This study shows that introduction of an electronegative substituent is quite promising to improve the antioxidant properties of imidazolylpolyphenols through the hydrogen transfer mechanism.

### 2.3. Evaluation of the Reaction Half-Life (t_1/2_) by Using Potentiometric Method

The kinetic features of the interaction of biologically active compounds are an important indicator, on the one hand, in terms of pharmacokinetics, speed and effectiveness of action, and on the other hand, shelf life.

Half-reaction periods (t_1/2_) for the studied compounds reacting with the oxidizing agent were determined by the potentiometric method as the time at which 50% of the obtained antioxidant capacity (AOC_1/2_, M-eq) is recorded. The data for further analysis were calculated from kinetic curves [41,55]. The half-reaction period is inversely proportional to the rate of the chemical reaction between the studied compound and the oxidizing agent, therefore, it is one of the indicators of reactivity. Values of half-reaction periods are given in Table 4.

Table 4 shows that the conjugation of phenylimidazole and polyphenol fragments has different effects on the half-reaction periods relative to the initial phenols. A decrease in value of the half-reaction period is observed in the case of imidazolylphloroglucinol; on the contrary, a slight increase of the half-reaction period, as well as a decrease of the interaction rate with the oxidant are the features observed for imidazolylpyrogallols and imidazolylhydroxyquinols. Additionally, according to the values of the half-reaction periods, all compounds can be classified as fast-acting antioxidants.

## 3. Materials and Methods

### 3.1. Apparatus

Voltammetric measurements were performed using the μAUTOLAB Type III potentiostat/galvanostat (Metrohm Autolab, Utrecht, Holland). A glassy carbon electrode (Metrohm Autolab, Utrecht, Holland) was used as a working electrode, a silver chloride (Metrohm Autolab, Utrecht, Holland) electrode was used as a reference electrode, and a glassy carbon rod (Metrohm Autolab, Utrecht, Holland) was used as an auxiliary electrode.

Potentiometric measurements were carried out by using the pH-meter “Expert-pH” (LLC “Econics-Expert”, Moscow, Russia) with the function of measuring EMF and RS-232 interface. The measurements were taken by means of an EPV-1 redox-platinum electrode and an EVL-1M silver-silver chloride electrode (Ag/AgCl/3 M KCl) (Gomelsky ZIP, Gomel, Belarus).

Thermostatted measurements (37 °C) were carried out with the use of a LOIP LT-205a circulating thermostat (JSC “LOIP”, Saint Petersburg, Russia).

Spectrophotometric measurements were carried out using an Evolution 201 UV-Visible Spectrophotometer (Thermo scientific, Waltham, MA, USA). The optical density of the solution was measured by the Folin assay at a wavelength of 765 nm in glass cuvettes l = 10 mm.

### 3.2. Reagents and Chemicals

The following reagents were used: KH_2_PO_4_, Na_2_HPO_4_·12H_2_O (Reachim, Moscow, Russia), reagent grade p.a. (buffer system); 2,2-azobis (2-methylpropionamidine) dihydrochloride (AAPH) (CAS: 2997-92-4, Sigma-Aldrich, St. Louis, MO, USA), reagent grade puriss.; phoroglucinol, pyrogallol, hydroxyquinol, gallic acid, and ascorbic acid (Sigma-Aldrich, St. Louis, MO, USA).

Solutions of the polyphenols and imidazole-derived polyphenols were prepared with distilled deionized water.

The antioxidant capacity was determined in a phosphate buffer solution (PBS) with pH 7.4. Redox transformations were investigated in 0.1 M KCl.

### 3.3. Experimental

Nuclear Magnetic Resonance (NMR) spectra were recorded on Bruker Avance II (400 MHz) spectrometers (Bruker, Billerica, MA, USA). All ^1^H-NMR experiments were reported in δ units, parts per million (ppm), and were measured relative to residual chloroform DCCl_3_ (7.26 ppm) or DMSO (2.50 ppm) signals in the deuterated solvent. All ^13^C-NMR spectra were reported in ppm relative to CDCl_3_ (77.16 ppm) or DMSO-*d*_6_ (39.52 ppm) and all spectra were obtained with ^1^H decoupling. All coupling constants *J* were reported in Hertz (Hz). The following abbreviations were used to describe peak splitting patterns (s = singlet, d = doublet, t = triplet, dd = doublet of doublet, m = multiplet, and br s = broad singlet). The mass spectra were recorded on a mass spectrometer SHIMADZU GCMS-QP2010 Ultra (SHIMADZU, Kyoto, Japan) with sample ionization by electron impact (EI). The IR spectra were recorded using a Fourier-transform infrared spectrometer (Bruker Corporation, 40 Manning Rd, Billerica, MA, USA) equipped with a diffuse reflection attachment. The elemental analysis was carried out on a Perkin Elmer Instrument (PerkinElmer, Waltham, MA, USA) equipped with CHN PE 2400 II analyzer. The course of the reactions was monitored by TCL on 0.25 mm silica gel plates (60F 254, MACHEREY-NAGEL Inc, 924 Marcon Blvd, Allentown, PA 18109, USA).

AcCl, phloroglucinol, pyrogallol, and hydroxyquinol were purchased and used as received.

2,2-Dimethyl-4-phenyl-2*H*-imidazole 1-oxide, 2,2-Dimethyl-4-(4-nitrophenyl)-2*H*-imidazole 1-oxide [59], 2,2-Dimethyl-4-(*p*-tolyl)-2*H*-imidazole 1-oxide, 2,2-Dimethyl-4-(4-(trifluoromethoxy)phenyl)-2*H*-imidazole-1-oxide [47], 4-(4-bromophenyl)-2,2-dimethyl-2*H*-imidazole 1-oxide [48] as starting material were prepared according to the literature procedures.

4-(2,2-dimethyl-5-(*p*-tolyl)-2*H*-imidazol-4-yl)benzene-1,2,3-triol hydrochloride (**1Pyr**), 4-(2,2-dimethyl-5-phenyl-2*H*-imidazol-4-yl)benzene-1,2,3-triol hydrochloride (**2Pyr**), 4-(5-(4-bromophenyl)-2,2-dimethyl-2*H*-imidazol-4-yl)benzene-1,2,3-triol hydrochloride (**3Pyr**), 4-(2,2-dimethyl-5-(4-(trifluoromethoxy)phenyl)-2*H*-imidazol-4-yl)benzene-1,2,3-triol hydrochloride (**4Pyr**), 5-(2,2-dimethyl-5-(*p*-tolyl)-2*H*-imidazol-4-yl)benzene-1,2,4-triol hydrochloride (**1Hyd**), 5-(2,2-dimethyl-5-phenyl-2*H*-imidazol-4-yl)benzene-1,2,4-triol hydrochloride (**2Hyd**), 5-(5-(4-bromophenyl)-2,2-dimethyl-2*H*-imidazol-4-yl)benzene-1,2,4-triol hydrochloride (**3Hyd**), 5-(2,2-dimethyl-5-(4-(trifluoromethoxy)phenyl)-2*H*-imidazol-4-yl)benzene-1,2,4-triol (**4Hyd**) were synthesized according to the previously reported procedure [44].

#### 3.3.1. General Procedure for the Synthesis of Hydrochloride Salt of Imidazole-Derived Polyphenolic Compounds **Im(1–5)Phl, Im5Pyr, Im5Hyd**

Acetyl chloride (1 mmol) was added to a vigorously stirred mixture of corresponding 2*H*-imidazole-1-oxide **ImNO(1–5)H** (1 mmol) and phenol **Phl, Pyr, Hyd** (1 mmol) in appropriated solvent at 0 °C. Then the resulting mixture was allowed to warm up to room temperature and stiffing continued for an additional 30 min. Then the resulting precipitate **Im(1–5)Phl, Im5Pyr, Im5Hyd** was filtered off and washed with the mixture of toluene/dichloromethane (50%/50% *w*/*v* 20 mL).

2-(2,2-Dimethyl-5-(*p*-tolyl)-2*H*-imidazol-4-yl)benzene-1,3,5-triol hydrochloride (**Im1Phl**). The general procedure was applied using acetone (4 mL) as solvent. Light-yellow solid. Yield: 0.94 mmol (326.0 mg, 94 %), mp = 210–215 °C. *R*_f_ 0.05 (hexane/EtOAc, 7:3). ^1^H-NMR (400 MHz, DMSO-*d*_6_): δ 10.37 (br s, 3H), 7.52 (d, *J* = 7.8 Hz, 2H); 7.23 (d, *J* = 7.7 Hz, 2H); 6.02 (s, 2H); 2.33 (s, 3H); 1.67 (s, 6H). ppm.^13^C{^1^H}-NMR (101 MHz, DMSO-*d*_6_): δ 164.5 (C); 163.9 (C); 161.7 (C); 159.1 (C); 140.9 (C); 129.4 (C); 128.97 (CH); 127.2 (CH); 97.4 (C); 94.7 (CH); 23.9 (CH_3_); 21.0 (CH_3_) ppm. IR (DRA): ν 3350, 3135, 1630, 1598, 1545, 1507, 1473, 1394, 1346, 1285, 1176, 1076, 827, 668, 606, 561 cm^−1^. MS (EI): *m*/*z* 310 [M]^+^. Anal. Calcd for C_18_H_19_ClN_2_O_3_: C, 62.34; H, 5.52; Cl, 10.22; N, 8.08; O, 13.84. Found: C, 62.11; H, 5.79; N, 7.78.

2-(2,2-Dimethyl-5-phenyl-2*H*-imidazol-4-yl)benzene-1,3,5-triol hydrochloride (**Im2Phl**). The general procedure was applied using acetone (4 mL) as solvent. Light-yellow solid. Yield: 0.95 mmol (315.9 mg, 95 %), mp = 195–200 °C. *R*_f_ 0.06 (hexane/EtOAc, 7:3). ^1^H-NMR (400 MHz, DMSO-*d*_6_): δ 10.29 (br s, 3H), 7.60 (d, *J* = 6.9 Hz, 2H); 7.50 (t, *J* = 7.3 Hz, 1H); 7.42 (t, *J* = 7.5 Hz, 2H); 6.01 (s, 2H); 1.66 (s, 6H) ppm.^13^C{^1^H}-NMR (101 MHz, DMSO-*d*_6_): δ 164.7, 164.3, 161.5, 159.3, 132.4, 130.9, 128.4, 127.0, 97.4, 95.4, 94.8, 23.9 ppm. IR (DRA): ν 3512, 3389, 3056, 1624, 1599, 1550, 1480, 1447, 1397, 1347, 1173, 1076, 823, 758, 694 cm^−1^. MS (EI): *m*/*z* 296 [M]^+^. Anal. Calcd for C_17_H_17_ClN_2_O_3_: C, 61.36; H, 5.15; Cl, 10.65; N, 8.42; O, 14.42. Found: C, 61.59; H, 5.34; N, 8.64.

2-(5-(4-Bromophenyl)-2,2-dimethyl-2*H*-imidazol-4-yl)benzene-1,3,5-triol hydrochloride (**Im3Phl**). The general procedure was applied using acetone (4 mL) as solvent. Light-yellow solid. Yield: 0.92 mmol (378.8 mg, 92 %), mp = 200–205 °C. *R*_f_ 0.08 (hexane/EtOAc, 7:3). ^1^H-NMR (400 MHz, DMSO-*d*_6_): δ 10.42 (br s, 3H), 7.64 (d, *J* = 8.3 Hz, 2H); 7.52 (d, *J* = 8.3 Hz, 2H); 6.06 (s, 2H); 1.67 (s, 6H). δ ppm.^13^C{^1^H}-NMR (101 MHz, DMSO-*d*_6_): δ 164.4 (C); 164.0 (C); 160.9 (C); 159.4 (C); 131.9 (C); 131.4 (CH); 129.1 (CH); 124.3 (C); 97.7 (C); 95.4 (C); 94.9 (CH), 23.8 (CH_3_) ppm. IR (DRA): ν 3430, 3060, 1628, 1589, 1474, 1401, 1346, 1277, 1173, 1073, 1041, 834, 665, 602, 553 cm^−1^. MS (EI): *m*/*z* 374 [M]^+^, 376 [M + 2]^+^. Anal. Calcd for C_17_H_16_BrClN_2_O_3_: C, 49.60; H, 3.92; Br, 19.41; Cl, 8.61; N, 6.80; O, 11.66. Found: C, 49.84; H, 4.12; N, 6.76.

2-(2,2-Dimethyl-5-(4-(trifluoromethoxy)phenyl)-2*H*-imidazol-4-yl)benzene-1,3,5-triol hydrochloride (**Im4Phl**). The general procedure was applied using acetone (4 mL) as solvent. Light-yellow solid. Yield: 0.87 mmol (362.6 mg, 87 %), mp = 205–210 °C. *R*_f_ 0.12 (hexane/EtOAc, 7:3). ^1^H-NMR (400 MHz, DMSO-*d*_6_): δ 10.30 (br s, 3H), 7.71 (d, *J* = 8.7 Hz, 2H); 7.43 (d, *J* = 8.4 Hz, 2H); 6.01 (s, 2H); 1.65 (s, 6H) ppm.^13^C{^1^H}-NMR (101 MHz, DMSO-*d*_6_): δ 163.7 (C); 160.9 (C); 159.2 (C); 149.8 (q, *J* = 1.9 Hz, C); 131.9 (C); 129.2 (CH); 120.6 (CH); 119.9 (q, *J* = 256.7 Hz, CF_3_); 98.0 (C); 95.9 (C); 94.8 (CH); 23.9 (CH_3_) ppm. ^19^F-NMR (376 MHz, DMSO-*d*_6_): δ -56.62 (s, 3F) ppm. IR (DRA): ν 3141, 2589, 1619, 1503, 1478, 1392, 1346, 1256, 1201, 1162, 1072, 830, 677, 598, 557 cm^−1^. MS (EI): *m*/*z* 380 [M]^+^. Anal. Calcd for C_18_H_16_ClF_3_N_2_O_4_: C, 51.87; H, 3.87; Cl, 8.51; F, 13.68; N, 6.72; O, 15.35. Found: C, 51.79; H, 4.07; N, 6.62.

2-(2,2-Dimethyl-5-(4-nitrophenyl)-2*H*-imidazol-4-yl)benzene-1,3,5-triol hydrochloride (**Im5Phl**). The general procedure was applied using acetone (4 mL) as solvent. Light-yellow solid. Yield: 0.88 mmol (332.5 mg, 88 %), mp = 175–180 °C. *R*_f_ 0.06 (hexane/EtOAc, 7:3). ^1^H-NMR (400 MHz, DMSO-*d*_6_): δ 10.25 (br s, 3H), 8.29 (d, *J* = 8.9 Hz, 2H); 7.81 (d, *J* = 8.8 Hz, 2H); 6.03 (s, 2H); 1.69 (s, 6H). ppm.^13^C{^1^H}-NMR (101 MHz, DMSO-*d*_6_): δ 164.6 (C); 163.9 (C); 160.3 (C); 159.6 (C); 148.4 (C); 139.3 (C); 128.4 (CH); 123.4 (CH); 98.3 (C); 95.5 (C); 94.9 (CH); 23.8 (CH_3_). ppm. IR (DRA): ν 3142, 2557, 1619, 1596, 1553, 1393, 1342, 1276, 1172, 1074, 853, 835, 711, 653, 551 cm^−1^. MS (EI): *m*/*z* 341 [M]^+^. Anal. Calcd for C_17_H_16_ClN_3_O_5_: C, 54.05; H, 4.27; Cl, 9.38; N, 11.12; O, 21.17. Found: C, 54.26; H, 4.50; N, 11.10.

4-(2,2-Dimethyl-5-(4-nitrophenyl)-2*H*-imidazol-4-yl)benzene-1,2,3-triol hydrochloride (**Im5Pyr**). The general procedure was applied using the hexachloroacetone/acetone (7 mL/1 mL) solvent mixture. Light-yellow solid. Yield: 0.91 mmol (310.3 mg, 91 %), mp = 155–160 °C. *R*_f_ 0.06 (hexane/EtOAc, 7:3). ^1^H-NMR (400 MHz, DMSO-*d*_6_): δ 8.33 (d, *J* = 8.9 Hz, 2H); 7.82 (d, *J* = 8.7 Hz, 2H); 6.53 (d, *J* = 8.7 Hz, 1H); 6.34 (d, *J* = 8.7 Hz, 1H); 1.64 (s, 6H) ppm.^13^C{^1^H}-NMR (101 MHz, DMSO-*d*_6_): δ 163.1 (C); 162.1 (C); 150.9 (C); 148.8 (C); 148.3 (C); 139.8 (C); 133.3 (CH); 129.7 (CH); 123.5 (CH); 121.6 (CH); 107.7 (C); 99.2 (C); 23.7 (CH_3_) ppm. IR (DRA): ν 3423, 2938, 1706, 1622, 1566, 1505, 1355, 1322,1280, 1098, 1041, 974, 861, 801, 768 cm^−1^. MS (EI): *m*/*z* 341 [M]^+^. Anal. Calcd for C_17_H_16_ClN_3_O_5_: C, 54.05; H, 4.27; Cl, 9.38; N, 11.12; O, 21.17. Found: C, 54.16; H, 4.02; N, 11.25.

5-(2,2-Dimethyl-5-(4-nitrophenyl)-2*H*-imidazol-4-yl)benzene-1,2,4-triol hydrochloride **(Im5Hyd**). The general procedure was applied using the hexachloroacetone/acetone (7 mL/1 mL) solvent mixture. Orange solid. Yield: 0.93 mmol (317.1 mg, 93 %), mp = 215–220 °C. *R*_f_ 0.04 (hexane/EtOAc, 7:3). ^1^H-NMR (400 MHz, DMSO-*d*_6_): δ 10.62 (br s, 3H), 8.38 (d, *J* = 8.8 Hz, 2H); 7.87 (d, *J* = 8.8 Hz, 2H); 6.85 (s, 1H); 6.52 (s, 1H); 1.72 (s, 6H) ppm.^13^C{^1^H}-NMR (101 MHz, DMSO-*d*_6_): δ 163.4 (C); 159.9 (C); 156.9 (C); 155.8 (C); 148.6 (C); 139.3 (C); 138.8 (C); 130.2 (CH); 123.7 (CH); 116.2 (CH); 103.6 (CH); 102.3 (C); 96.9 (C); 23.5 (CH_3_) ppm. IR (DRA): ν 3072, 1623, 1574, 1529, 1510, 1444, 1357, 1311, 1237, 1166, 1014, 910, 850, 722, 653 cm^−1^. MS (EI): *m*/*z* 341 [M]^+^. Anal. Calcd for C_17_H_16_ClN_3_O_5_: C, 54.05; H, 4.27; Cl, 9.38; N, 11.12; O, 21.17. Found: C, 53.93; H, 4.51; N, 11.17.

#### 3.3.2. General Procedure for the Synthesis of 2*H*-imidazoles **Im1–2**

A few spoons (~ 0.7–1.2 g) of Raney-Nickel were placed in distilled water (5 mL) followed by addition of saturated NaOH solution until hydrogen release stopped. Caution: this process is highly fire hazardous and explosive. Then, the mixture was filtered off, washed with water (3 mL × 15 mL) and EtOH (3 mL × 15 mL). The obtained activated nickel was added to the mixture of 2*H*-imidazole-*N*-oxide **ImNO(1–2)H** (2 mmol, 1 eq) in EtOH (15 mL) followed by addition of hydrazine hydrate (1.1 mmol, 0.55 equiv.). The reaction was refluxed in an oil bath at 80 °C for 4–6 h until the starting material disappeared (monitored by TLC). The resulting mixture was filtered through short columns (SiO_2_) with EtOH as eluent (50 mL) and concentrated under reduced pressure. The desired product **Im(1–2)H** was obtained by manual column chromatography (SiO_2_) with hexane/ethyl acetate 7/3 as eluent.

2,2-Dimethyl-4-(*p*-tolyl)-2*H*-imidazole **(Im1**). Brown solid. Yield: 1.44 mmol (267.8 mg, 72 %), mp = 60–65 °C. *R*_f_ 0.2 (hexane/EtOAc, 7:3). ^1^H-NMR (400 MHz, DCCl_3_): δ 8.27 (s, 1H), 7.80 (d, *J* = 7.9 Hz, 2H), 7.21 (d, *J* = 7.29 Hz, 2H), 2.33 (s, 3H), 1.49 (s, 6H) ppm.^13^C{^1^H}-NMR (101 MHz, DCCl_3_): δ 167.8, 163.2, 154.2, 141.8, 129.8, 128.2, 105.4, 23.9, 21.6 ppm. IR (DRA): ν 2959, 2930, 2861, 1718, 1609, 1455, 1410, 1269, 1217, 1163, 1114, 927, 820, 742, 582, 511 cm^−1^. MS (EI): *m*/*z* 186 [M]^+^. Anal. Calcd for C_12_H_14_N_2_: C, 77.38; H, 7.58; N, 15.04. Found: C, 77.67; H, 7.59; N, 15.28.

2,2-Dimethyl-4-phenyl-2*H*-imidazole **(Im2**). Brown oil. Yield: 1.6 mmol (275.2 mg, 80%), *R*_f_ 0.26 (hexane/EtOAc, 7:3). ^1^H-NMR (400 MHz, DCCl_3_): δ δ 8.34 (s, 1H), 7.99–7.95 (m, 2H), 7.51–7.45 (m, 3H), 1.56 (s, 6H) ppm.^13^C{^1^H}-NMR (101 MHz, DCCl_3_): δ 163.3, 153.9, 131.6, 131.3, 129.1, 128.2, 105.7, 23.9 ppm. IR (DRA): ν 3057, 2929, 2853, 1696, 1610, 1534, 1490, 1446, 1266, 1018, 953, 915, 851, 686, 567 cm^−1^. MS (EI): *m*/*z* 172 [M]^+^. Anal. Calcd for C_11_H_12_N_2_: C, 76.71; H, 7.02; N, 16.27. Found: C, 76.94; H, 7.22; N, 16.12.

### 3.4. Procedures

#### 3.4.1. Cyclic Voltammetry

Cyclic voltammograms were obtained on a glassy carbon electrode in 0.1 M KCl at a scan rate of 0.1 V/s. The position of the anodic oxidation peak (E_ox_) and the magnitude of the oxidation peak current (i_ox_) were determined.

#### 3.4.2. Potentiometric Assay of Determining Antioxidant Capacity

Determination of antioxidant capacity (AOC) was carried out by the potentiometric method using the oxidizing agent K_3_[Fe(CN)_6_]. The use of potassium hexacyanoferrate (III) as an oxidizing agent is justified both from a thermodynamic point of view and from the point of view of obtaining an optimal analytical signal [40,41,54,55,56]. An analytical signal, in this case, is the shift of the potential of the platinum electrode in the K_3_[Fe(CN)_6_]/K_4_[Fe(CN)_6_] system, which is observed when the analyzed sample is introduced (Figure S22). This shift is due to a change in the ratio of the system components as a result of the reaction:

b·[Fe(CN)_6_]^3−^ + AO → b·[Fe(CN)_6_]^4−^ + AO_Ox_ (1)

where AO—antioxidant, and AO_Ox_—antioxidant are oxidation products.

Antioxidant capacity (M-eq) was calculated using the formulas:

(2) $$\mathrm{AOC}=\frac{c_{\mathrm{Ox}}-\alpha c_{\mathrm{Red}}}{1+\alpha }\cdot n$$

(3) $$\alpha =(c_{\mathrm{Red}}/c_{\mathrm{Ox}})10^{(E_{2}-E_{1})F/2.3\mathrm{RT}}$$

where *c*_Ox_ is the concentration of K_3_[Fe(CN)_6_], M; *c*_Red_ is the concentration of K_4_[Fe(CN)_6_], M; E_1_ is the measured potential before the introduction of the analyzed sample, V; E_2_ is the measured potential after the introduction of the analyzed sample, V, n is the sample dilution degree.

The half-life period (t_1/2_) of the studied compounds with the oxidizing agent was determined by the potentiometric method from the kinetic curve of the AOC change on time, as the time during which 50% of the obtained antioxidant capacity was recorded (AOC_1/2_ = AOC/2 mol/L) [41,55].

### 3.5. Potentiometric Assay of Determining Antiradical Capacity

The basis for the definition of total antiradical capacity (ARC) is a regular change in the redox potential of the reaction of antioxidants with peroxyl radicals generated during the thermal decomposition of 2,2′-azobis (2-amidinopropane) dihydrochloride (Figure S23) [40,41,56,57].

The determination of the antiradical capacity (ARC) was carried out according to the formula:

ARC = W_i_·τ (4)

where ARC is the antiradical capacity, mol-eq/L; and τ is the induction period, s.

The number of equivalents in the AO molecule usually corresponds to the number of functional antioxidant groups involved in the inhibition of one radical chain.

The induction period was determined as the time from the introduction of the antioxidant into the initiator solution to the point corresponding to the maximum rate of change of the potential (dE/dt)_max_, which is defined as the maximum of the derivative function in the dependence of the redox potential on time.

Studies were performed at pH = 7.4 in PBS and 37 °C.

### 3.6. Folin Assay

The total content of polyphenols was determined in accordance with the use of the Folin–Ciocalteu reagent (10 g Na_2_WO_4_·2H_2_O, 2.5 g Na_2_MoO_4_·2H_2_O, 5 mL 85% H_3_PO_4_, 10 mL 35% HCl, 15 g Li_2_SO_4_·H_2_O, 100 mL H_2_O) [43]. A 10% solution was used. The Folin–Ciocalteu reagent contains phosphoric-tungstic acids, which are reduced by OH-groups of the phenolic compound to form tungsten blue (λ_max_ = 765 nm). Gallic acid was used as a standard.

### 3.7. DPPH Assay

The scavenging ability in relation to stable radical DPPH^•^ was determined by an optical method using the reaction [42]:

DPPH^•^ + AO-H→DPPH-H + AO^•^ (5)

The stable radical DPPH^•^ has a strong violet band with an absorption maximum at 518 nm. The DPPH assay was assessed from the absorption decrease at the given wavelength. The DPPH solution was prepared using EtOH (96%). The colour intensity of the DPPH solution (0.2 mmol/dm^3^) decreased after adding an aliquot of the investigated sample to it. Ascorbic acid was used as a standard (0.01–0.05 mmol/dm^3^).

### 3.8. Data Treatment

Five replications of the measurements were performed. Statistical evaluation was performed at the significance level of 5%. All data were expressed as X ± ΔX, where X is the average value and DX is the confidence interval. Calculation of correlations was performed using the Pearson criterion.

## 4. Conclusions

The synthesis of bifunctional imidazolylphloroglucinols bearing both electron-donating and electron-withdrawing substituents in the aryl fragment along with a variety in the arrangement of hydroxy groups in the polyphenol moiety (derivatives of phloroglucinol, pyrogallol and hydroxyquinol) was carried out using the pot and atom economy methodology of C-H functionalization of non-aromatic azaheterocyclic substrates that was realized as reactions of nucleophilic substitution of hydrogen (S_N_^H^) in 2*H*-imidazole 1-oxides. It is worth noting that the reaction of *2H*-imidazole-*N*-oxides ImNO(1–5)H with polyphenols Phl, Pyr, Hyd afforded a series of previously unknown 2*H*-imidazole-derived phenolic compounds, such as Im(1–5)Phl, Im5Pyr, Im5Hyd in good-to-excellent yields (87–95%). To estimate how the arylimidazole moiety affects antiradical properties of bifunctional molecular systems, the corresponding reduced forms of some 2*H*-imidazole-*N*-oxides Im(1–2)H for the first time. All the synthesized compounds were fully characterized by the data of ^1^H, ^13^C-NMR and IR-spectroscopy, mass-spectrometry, and elemental.

The expediency of using the integrated approach to study antioxidant properties from the standpoint of mechanisms of biological transformations was substantiated. The complex approach included the methods based on individual mechanisms and their combinations: electron-transfer (ET), hydrogen atom transfer (HAT), and chelating mechanism.

The data of the cyclic voltammetry have shown that the conjugation of imidazole and polyphenol fragments leads to a shift of the oxidation peak for imidazolylpolyphenols into the positive region, which can be attributed to a decrease in the hydrogen atom mobility and a more complicated electron transfer process. The latter finally results in a lower activity via both electron transfer and chelation mechanisms regardless of the structure of the phenolic fragment. Additionally, the structure of the phenol substituent in imidazolylpolyphenols has a significant effect on their antiradical properties. There is, however, no effect of conjugation on the antiradical capacity of pyrogallol derivatives. Meanwhile, the conjugation enhances the antiradical capacity of imidazolylphloroglucinols and decreases that of oxyhydroquinone derivatives. Imidazole derivatives bearing the electronegative nitro group proved to have the highest values for their antioxidant and antiradical capacity, thus suggesting that electron-withdrawing functionalities can improve antioxidant properties of imidazolylpolyphenols.

In this work, we first showed that in the case of phloroglucinol derivatives, the conjugation effect results in a significant increase in the antiradical capacity (ARC) for a whole range of compounds compared to the starting phenol. In particular, the conjugation of phloroglucinol moiety with an Im5 fragment was demonstrated to lead to a significant increase in ARC from 2.26 to 5.16 (10^4^ mol-eq /L). This means that the considered family of compounds is capable of exhibiting an activity realized by the method of transferring a hydrogen atom, which exceeds the activity of known natural polyphenolic compounds, and one molecule Im5Phl is capable of quenching 5 radical oxidative chains. The latter could be considered as a valuable result in the development of bifunctional compounds realizing their biological activity via various mechanisms of biological action to be of particular interest in the modern pharmacology and medicinal chemistry.

## Figures and Tables

**Table 1:** Structures of molecular systems.

Polyphenol Scaffold Imidazole Scaffold	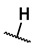	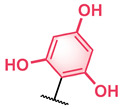	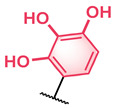	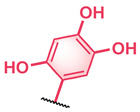
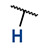		**Phl ^a^**	**Pyr ^a^**	**Hyd ^a^**
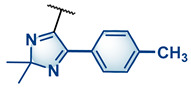	**Im1H** (72%)	**Im1Phl** (94%)	**Im1Pyr** (85%) ^b^	**Im1Hyd** (93%) ^b^
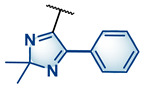	**Im2H** (80%)	**Im2Phl** (95%)	**Im2Pyr** (75%) ^b^	**Im2Hyd** (88%) ^b^
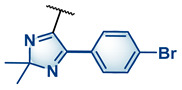	**-**	**Im3Phl** (92%)	**Im3Pyr** (90%) ^b^	**Im3Hyd** (91%) ^b^
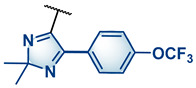	**-**	**Im4Phl** (87%)	**Im4Pyr** (87%) ^b^	**Im4Hyd** (90%) ^b^
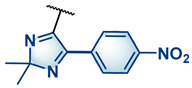	**-**	**Im5Phl** (88%)	**Im5Pyr** (91%)	**Im5Hyd** (93%)

a: from commercial source, b: yield according to the literature procedure [42].

**Table 2:** Potentials and currents of oxidation peaks for phenols and imidazolylpolyphenols.

Polyphenol	Imidazolylpolyphenols	E_ox_, V	i_ox_, 10^6^ A
Phloroglucinol	**Phl**	0.64	2.80
	**Im1Phl**	1.02	3.27
	**Im2Phl**	1.02	1.85
	**Im3Phl**	0.99	4.69
	**Im4Phl**	1.02	4.45
	**Im5Phl**	0.98	5.09
Pyrogallol	**Pyr**	0.18 0.60	2.90 1.63
	**Im1Pyr**	0.57 1.09	6.61 1.93
	**Im2Pyr**	0.45 1.00	8.78 3.10
	**Im3Pyr**	0.45 0.98	7.82 3.23
	**Im4Pyr**	0.52 1.03	9.35 3.51
	**Im5Pyr**	0.18 0.64	5.59 7.00
Hydroxyquinol	**Hyd**	−0.02 1.08	9.80 2.20
	**Im1Hyd**	0.36 1.11	10.41 11.41
	**Im2Hyd**	0.54 1.20	7.26 6.64
	**Im3Hyd**	0.38 1.12	8.62 7.97
	**Im4Hyd**	0.38 1.15	14.40 12.37
	**Im5Hyd**	0.14 1.20	1.27 6.46

**Table 3:** Antioxidant properties of phenols and imidazolylpolyphenols (10^4^ mol-eq/dm^3^, 25 °C, *n* = 5, *p* = 0.95).

Substrates	AOC		ARC		Folin Assay		DPPH Assay	
	10^4^ mol-eq/dm^3^	RSD (%)	10^4^ mol-eq/dm^3^	RSD (%)	C(GA) 10^4^ mol-eq/dm^3^	RSD (%)	C(AA) 10^4^ mol-eq/dm^3^	RSD (%)
**Phl**	2.84 ± 0.23	8.1	2.26 ± 0.04	2.2	-	-	1.22 ± 0.02	1.4
**Pyr**	5.15 ± 0.11	1.9	2.84 ± 0.03	3.2	2.08 ± 0.02	0.9	1.47 ± 0.03	1.2
**Hyd**	1.92 ± 0.04	1.9	1.89 ± 0.02	0.9	0.74 ± 0.01	1.3	1.40 ± 0.01	1.5
**Im1Phl**	1.95 ± 0.04	1.9	4.01 ± 0.08	1.9	-	-	0.78 ± 0.01	1.4
**Im2Phl**	2.74 ± 0.08	3.1	4.02 ± 0.16	3.8	-	-	0.71 ± 0.03	3.3
**Im3Phl**	1.89 ± 0.02	0.9	*		-	-	-	-
**Im4Phl**	1.87 ± 0.04	2.1	*		-	-	0.69 ± 0.01	2.4
**Im5Phl**	1.88 ± 0.08	4.1	5.16 ± 0.17	2.9	-	-	0.70 ± 0.02	2.1
**Im1Pyr**	2.24 ± 0.09	3.8	2.15 ± 0.02	0.9	0.50 ± 0.05	9.1	1.17 ± 0.02	2.1
**Im2Pyr**	2.46 ± 0.02	1.2	1.51 ± 0.02	1.9	0.44 ± 0.004	1.1	1.17 ± 0.01	1.2
**Im3Pyr**	1.76 ± 0.07	3.9	2.52 ± 0.07	3.2	0.40 ± 0.02	5.5	0.93 ± 0.04	4.2
**Im4Pyr**	1.94 ± 0.04	1.7	2.97 ± 0.03	0.8	0.41 ± 0.03	7.9	1.22 ± 0.01	1.1
**Im5Pyr**	4.19 ± 0.34	7.8	1.26 ± 0.06	4.7	0.61 ± 0.07	10.6	1.22 ± 0.01	1.1
**Im1Hyd**	0.98 ± 0.03	2.7	0.41 ± 0.01	1.6	0.68 ± 0.05	6.9	1.12 ± 0.02	1.9
**Im2Hyd**	0.75 ± 0.05	6.7	0.40 ± 0.02	3.8	0.78 ± 0.05	6.1	1.06 ± 0.01	1.3
**Im3Hyd**	0.72 ± 0.01	1.7	0.81 ± 0.02	2.9	1.15 ± 0.06	5.1	1.12 ± 0.07	5.9
**Im4Hyd**	1.58 ± 0.03	1.8	1.13 ± 0.06	4.6	0.75 ± 0.09	11.0	0.95 ± 0.02	1.9
**Im5Hyd**	2.78 ± 0.25	8.8	0.34 ± 0.06	2.3	0.92 ± 0.09	10.1	1.22 ± 0.00	0

* Could not be determined due to the nonclassical form of the kinetic dependence of the potential on time.

**Table 4:** Values of half-reaction periods for the studied compounds (*n* = 5, *p* = 0.95).

Substance	t_1/2_ (s)	RSD (%)
Phloroglucinol	133 ± 6	4.8
Pyrogallol	5.2 ± 0.3	4.6
Hydroxyquinol	1.3 ± 0.1	7.6
**Im1Phl**	42 ± 2	4.7
**Im2Phl**	86 ± 3	4.0
**Im3Phl**	52 ± 2	4.6
**Im4Phl**	46 ± 3	8.1
**Im5Phl**	73 ± 7	9.2
**Im1Pyr**	9.2 ± 0.3	2.4
**Im2Pyr**	5.5 ± 0.4	6.9
**Im3Pyr**	15.3 ± 0.7	4.7
**Im4Pyr**	7.2 ± 0.1	3.0
**Im5Pyr**	11.3 ± 0.64	5.9
**Im1Hyd**	4.5 ± 0.2	4.2
**Im2Hyd**	6.4 ± 0.2	3.9
**Im3Hyd**	5.5 ± 0.3	4.7
**Im4Hyd**	11.1 ± 0.5	4.8
**Im5Hyd**	4.5 ± 0.1	2.3

## Data Availability

The data presented in this study are available on request from the corresponding author and co-authors.

## Supplementary Materials

Copies of NMR spectra, data on correlation between the position of the oxidation peak for pyrogallol and hydroxyquinol derivatives, antioxidant capacity assay, and antiradical capacity assay.

## Sample Availability

Samples of the compounds 2*H*-imidazole-derived phenolic compounds are available from the authors.
