# Supplementary material for: Design and Antioxidant Properties of Bifunctional 2H-Imidazole-Derived Phenolic Compounds—A New Family of Effective Inhibitors for Oxidative Stress-Associated Destructive Processes
Source: Molecules. 2021 Oct 29;26(21):6534. doi: 10.3390/molecules26216534 (PMC8588064; doi:10.3390/molecules26216534)
Supplement: Supplementary file 1 [file molecules-26-06534-s001.zip › molecules-1397620-supplementary.pdf]

Design and antioxidant properties of bifunctional 2*H*-imidazole-derived phenolic compounds - a new family of effective inhibitors for oxidative stress-associated destructive processes

Gerasimova E.L.[1], Gazizullina E.R.[1], Borisova M.V.[1], Igdisanova D.I.[1], Nikiforov E.A.[1], Moseev T.D.[1], Varaksin M.V.[1,2], Chupakhin O.N.[1,2], Charushin V.N.[1,2], Ivanova A.V.[1]

[1] *Ural Federal University, 19 Mira Str., 620002 Ekaterinburg, Russia*
[2] *Postovsky Institute of Organic Synthesis, Ural Branch of the Russian Academy of Sciences, 22 S. Kovalevskaya Str., 620990 Ekaterinburg, Russia*

Table of contests

1. Copies of NMR spectra S2
2. Correlation between the position of the oxidation peak for pyrogallol and hydroxyquinol derivatives S12
3. Antioxidant capacity assay S13
4. Antiradical capacity assay S14

1. Copies of NMR spectra

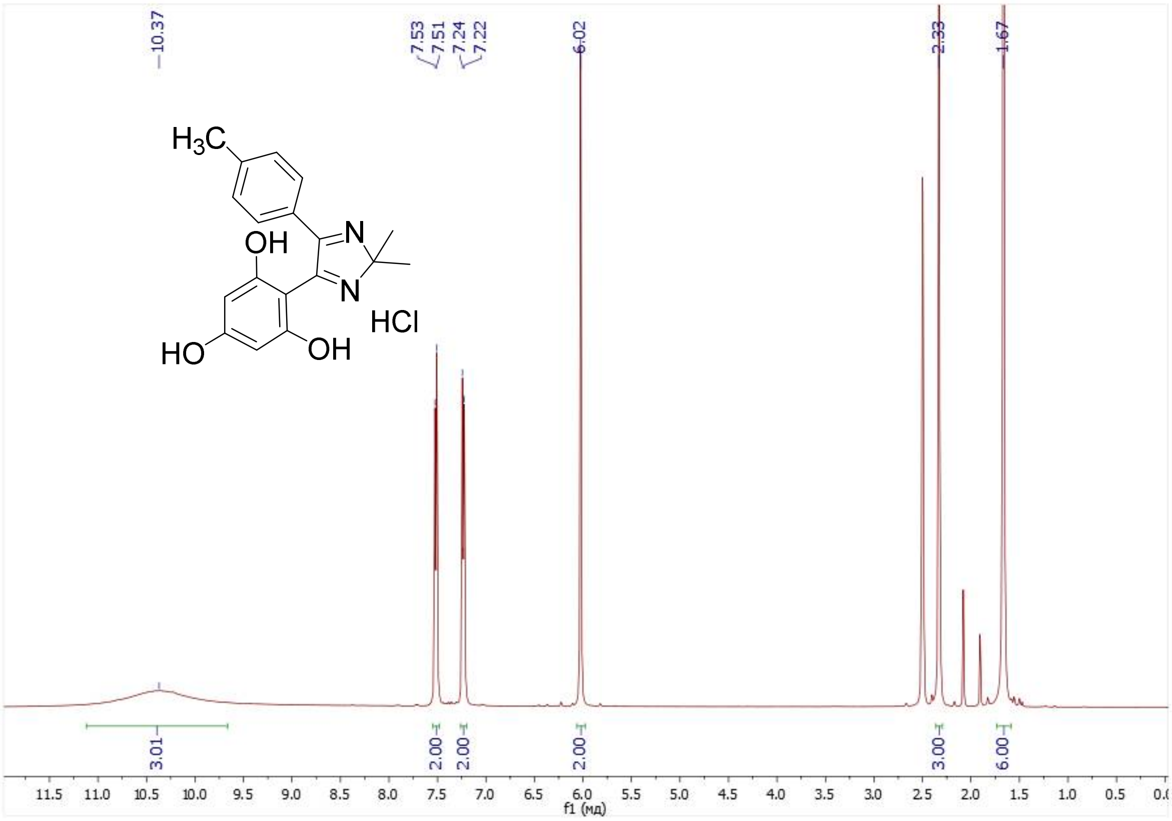

Figure S1. ^{1}H NMR spectrum (400 MHz, DMSO-d_6) of compound **Im1Phl**

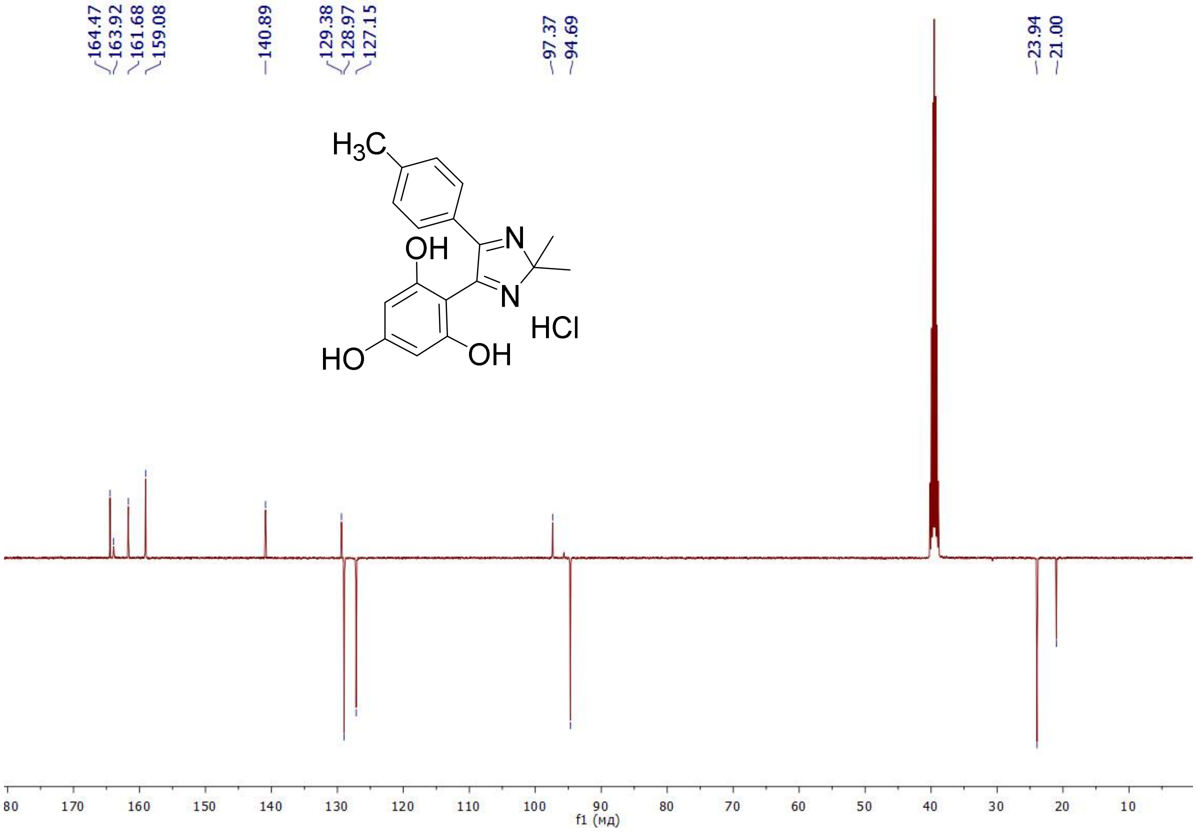

Figure S2. $^{13}C\{^{1}H\}$ NMR spectrum (101 MHz, DMSO-d_6) of compound **Im1Phl**

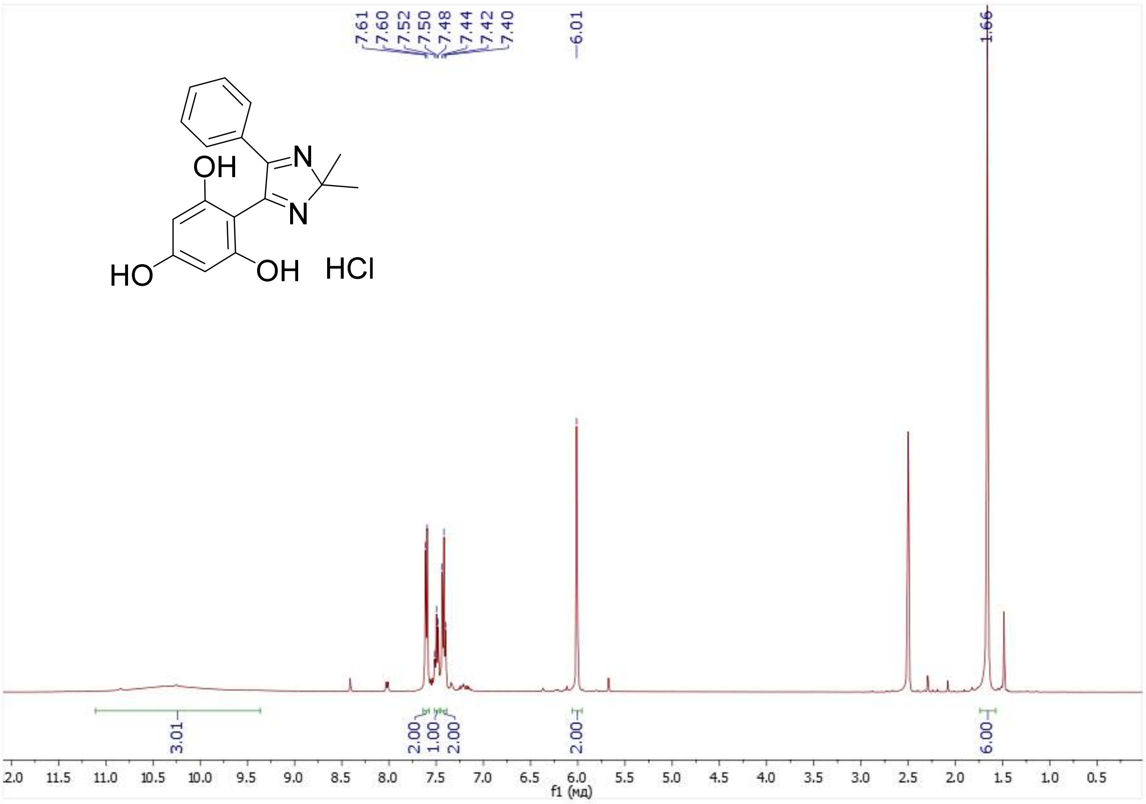

Figure S3. ^{1}H NMR spectrum (400 MHz, DMSO-d6) of compound **Im2Phl**

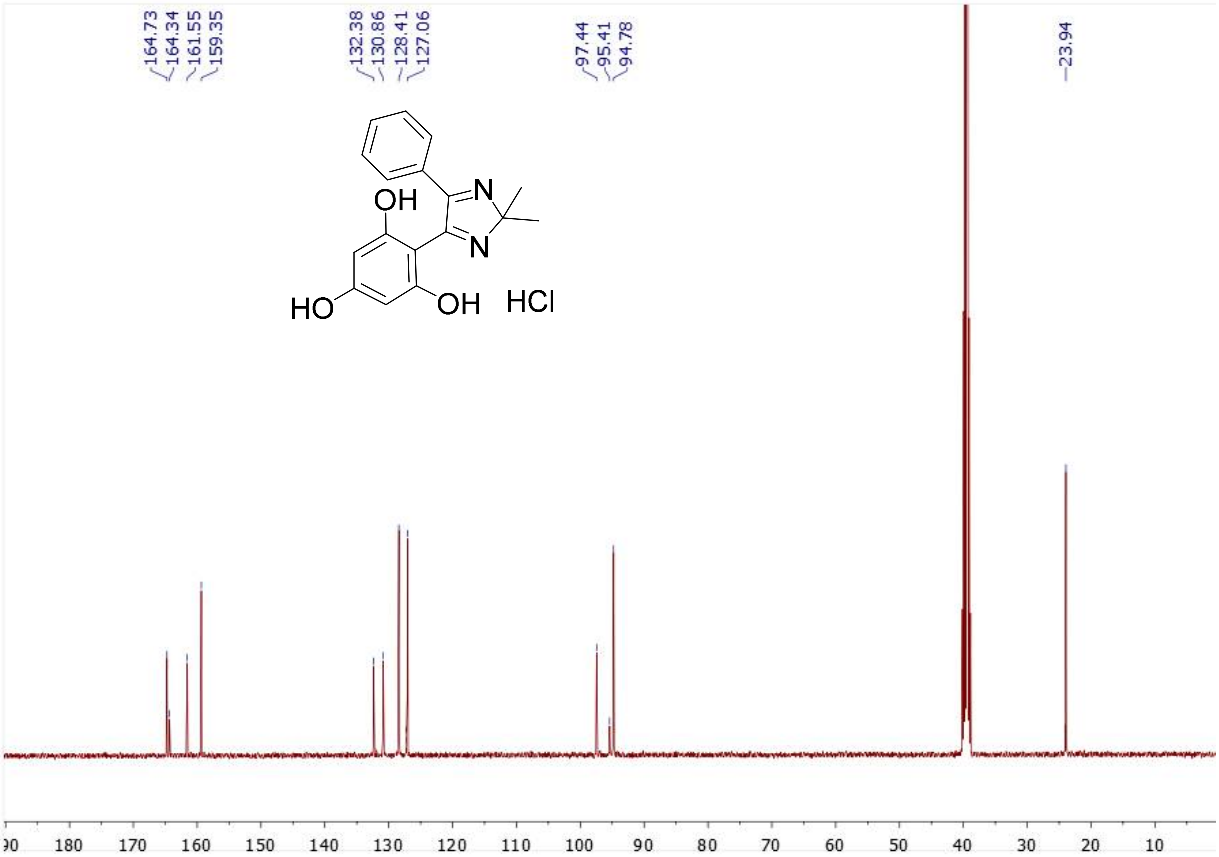

Figure S4. ^{13}C{^{1}H} NMR spectrum (101 MHz, DMSO-d6) of compound **Im2Phl**

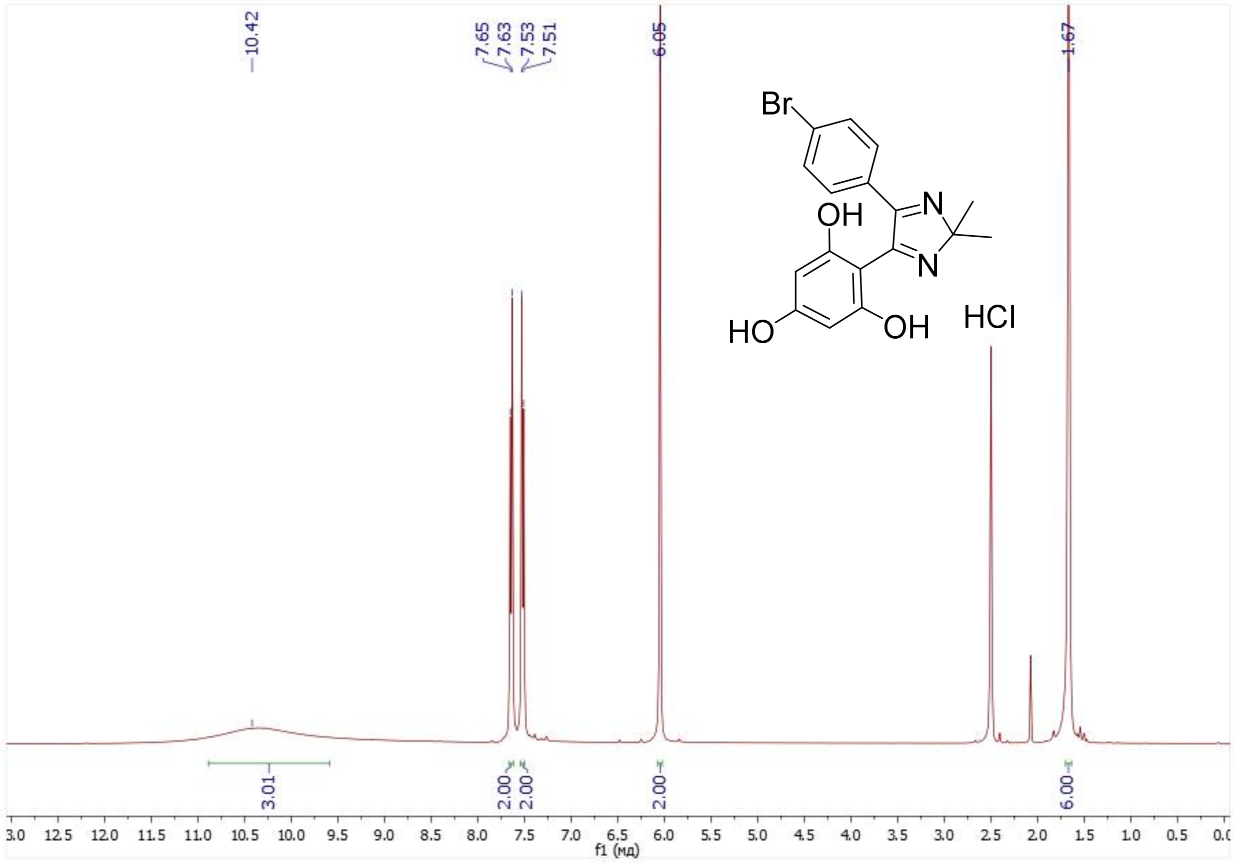

Figure S5. ^{1}H NMR spectrum (400 MHz, DMSO-d_6) of compound **Im3Phl**

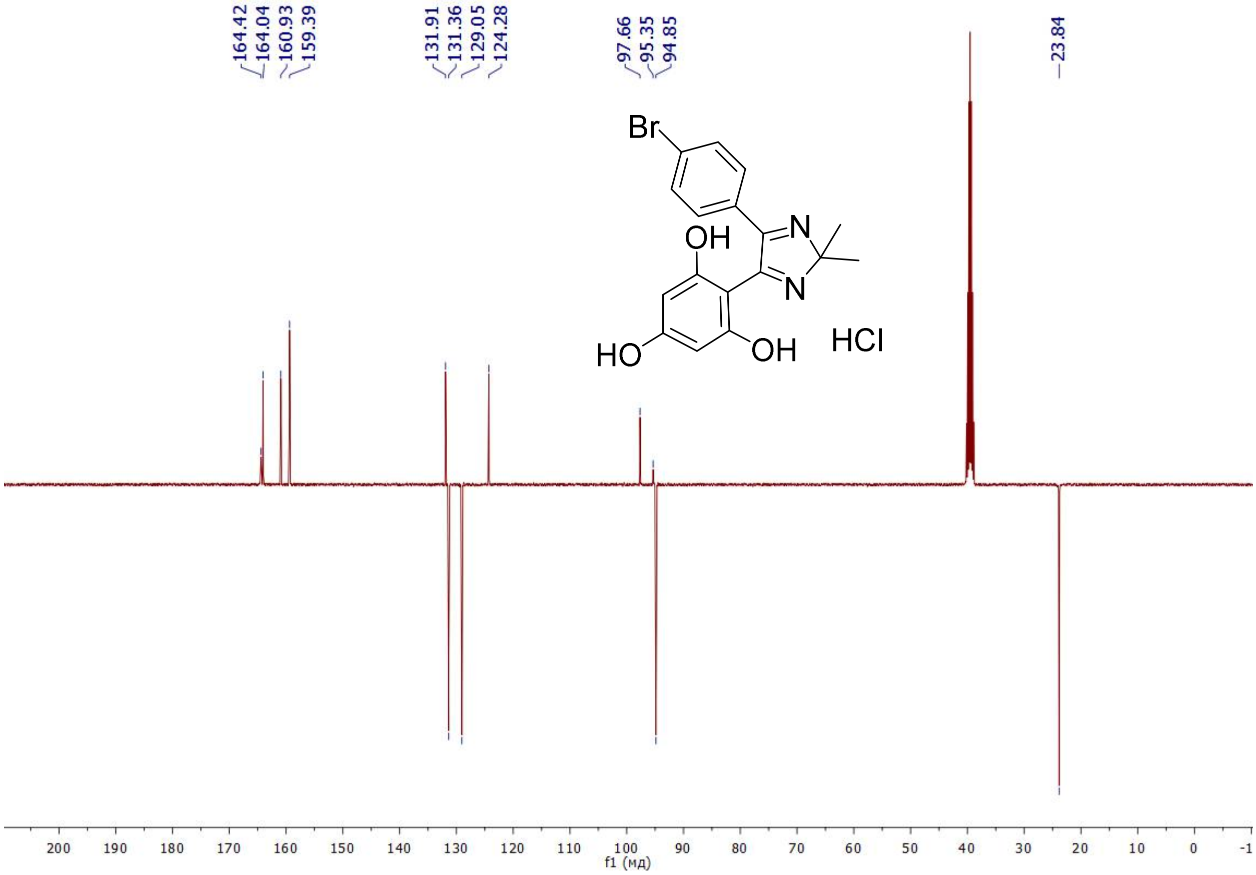

Figure S6. $^{13}C\{^{1}H\}$ NMR spectrum (101 MHz, DMSO-d_6) of compound **Im3Phl**

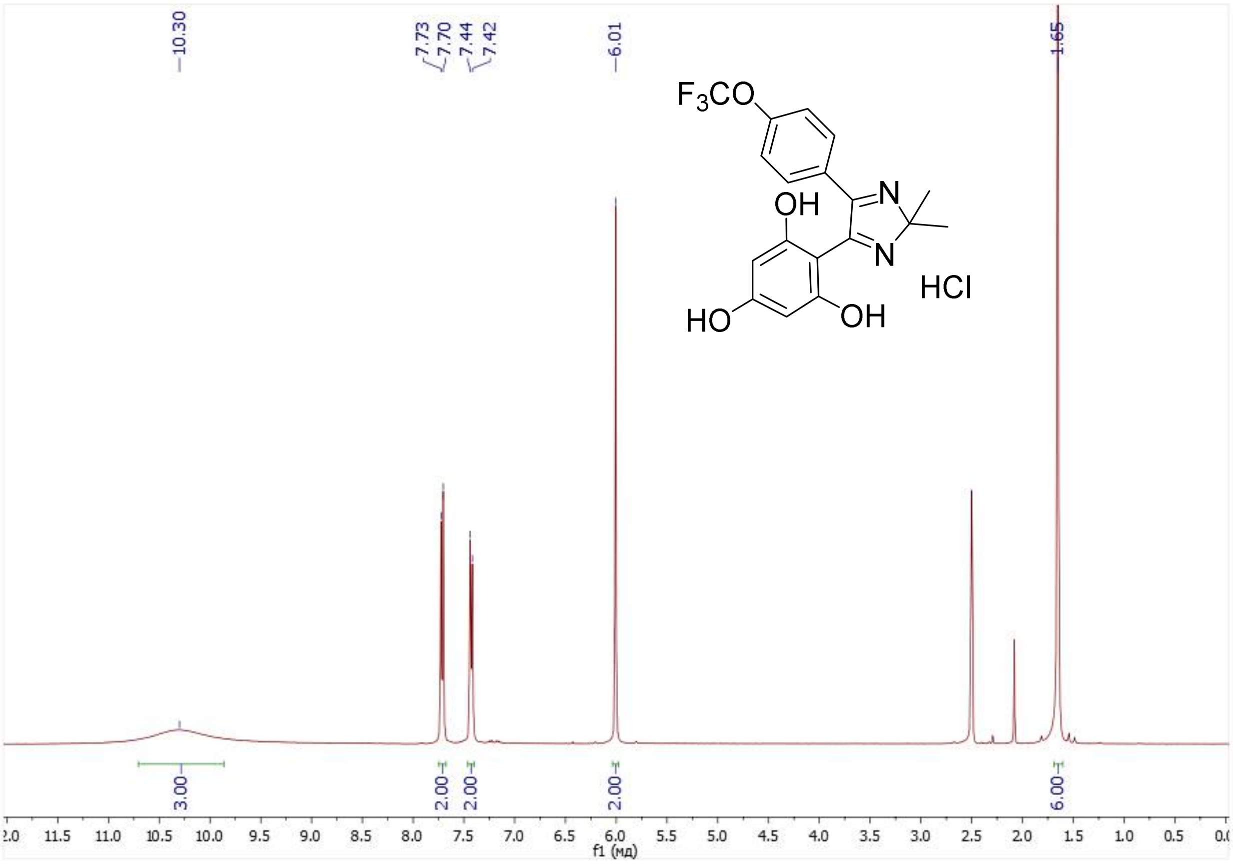

Figure S7.^{1}H NMR spectrum (400 MHz, DMSO-d$_6$) of compound **Im4Phl**

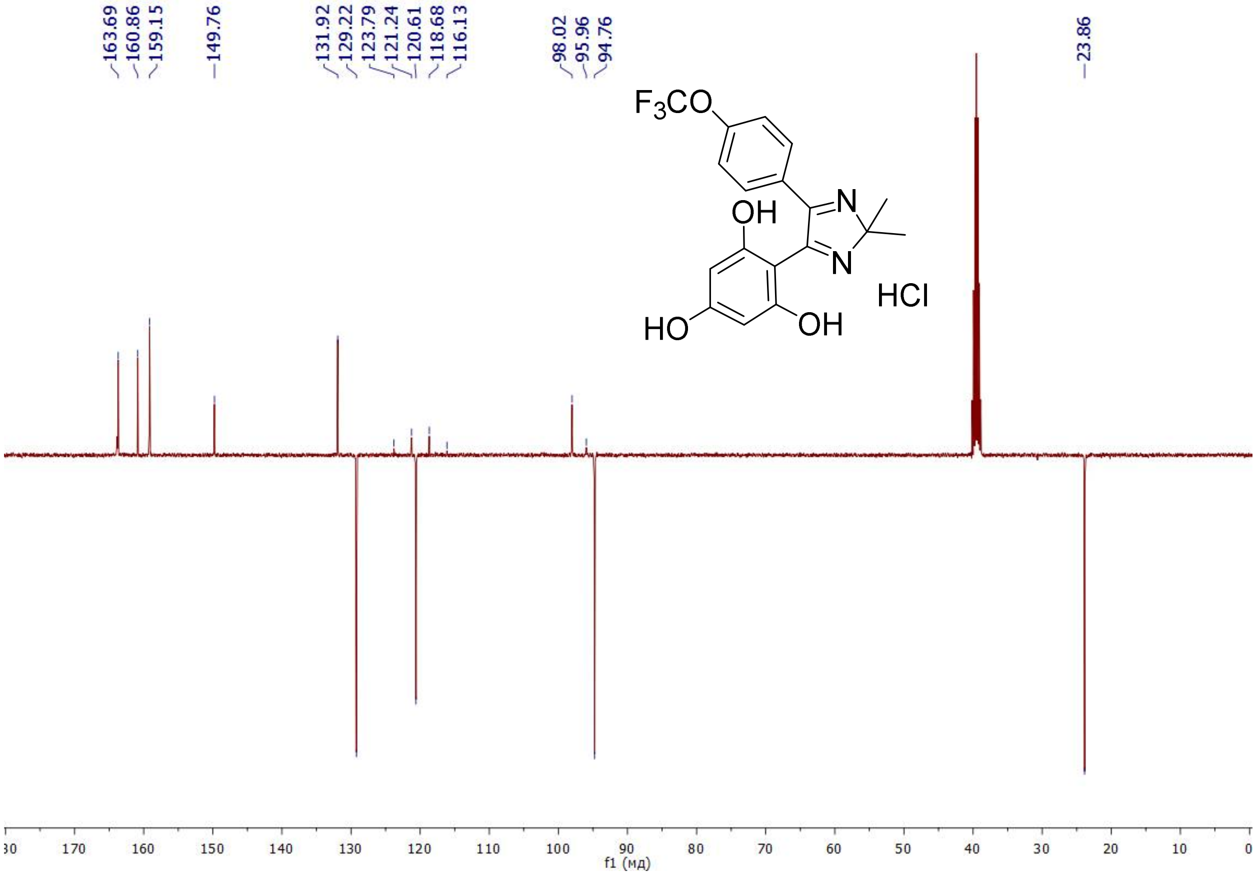

Figure S8. $^{13}C\{^{1}H\}$ NMR spectrum (101 MHz, DMSO-d$_6$) of compound **Im4Phl**

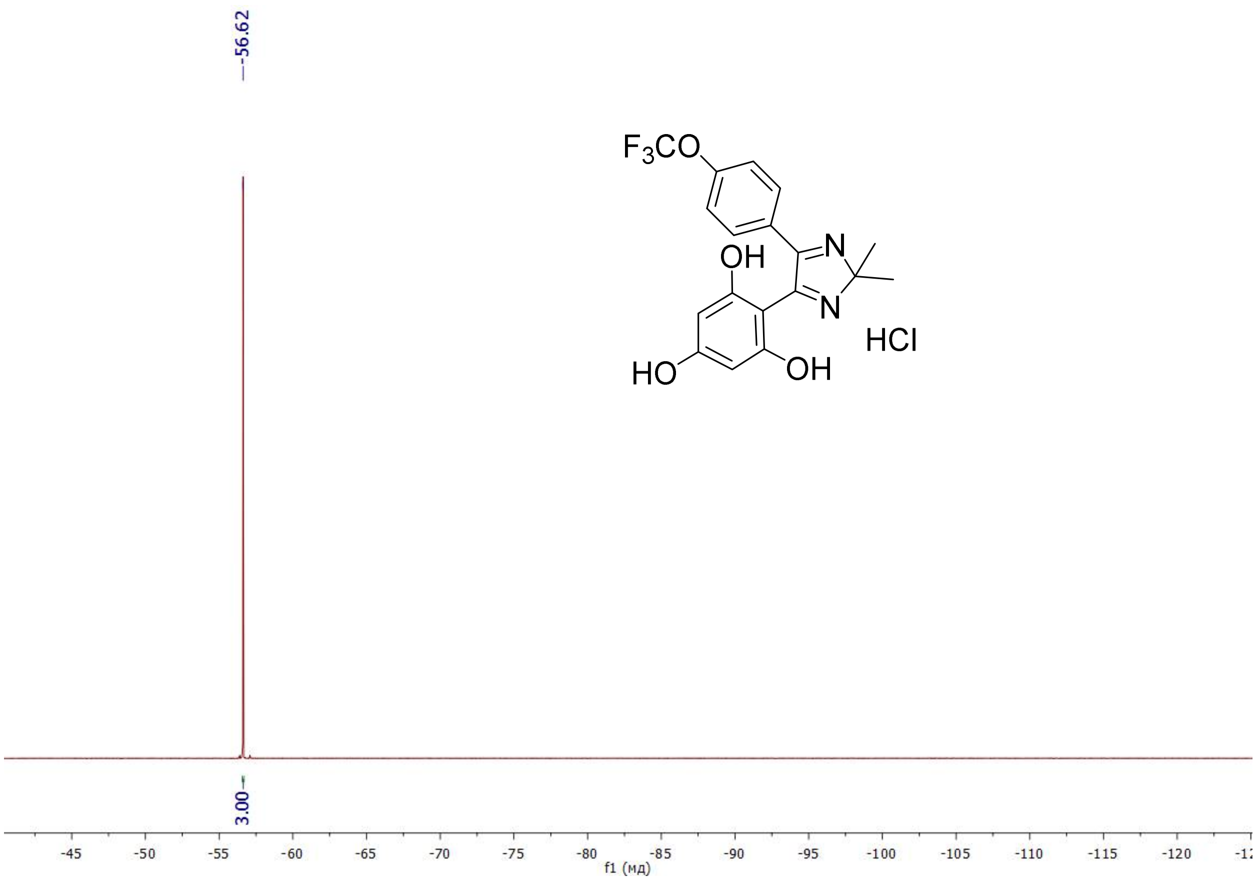

Figure S9. ^{19}F NMR spectrum (376 MHz, DMSO-d_6) of compound **Im4Phl**

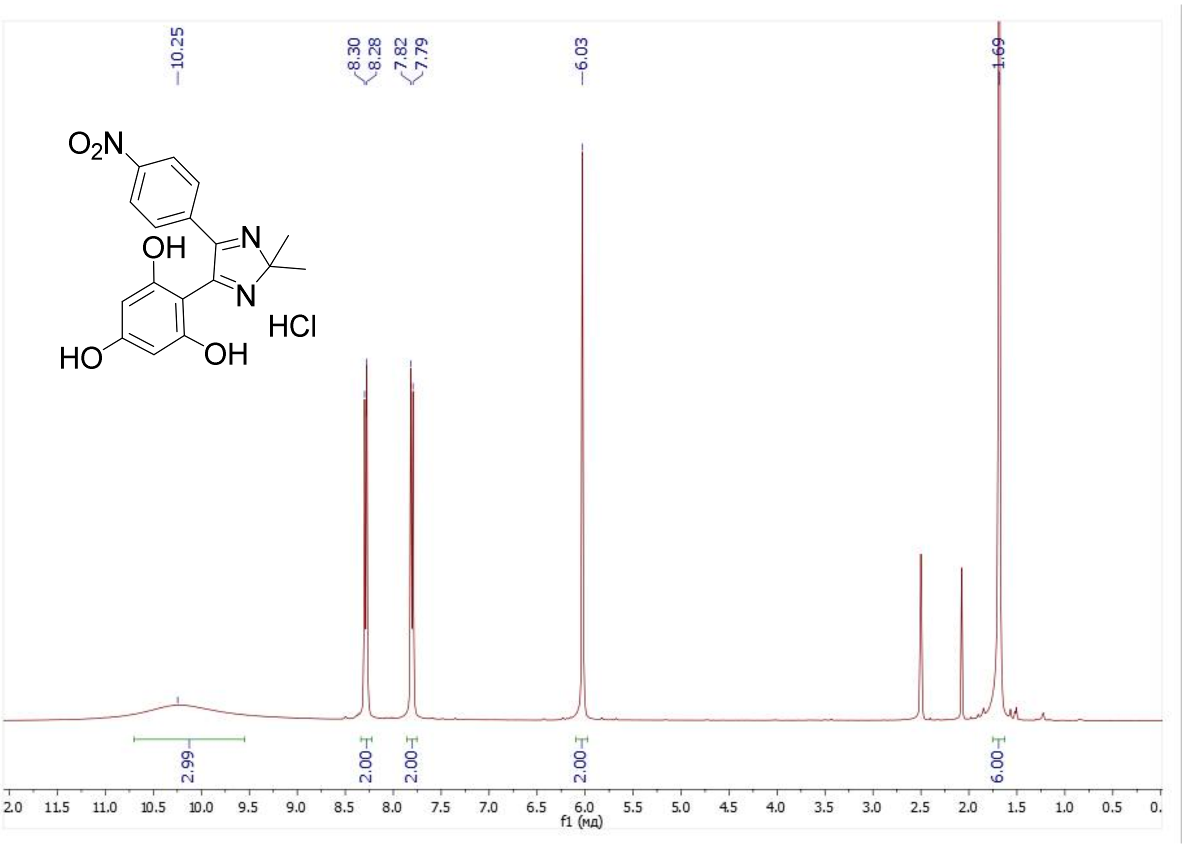

Figure S10. ^{1}H NMR spectrum (400 MHz, DMSO-d_6) of compound **Im5Phl**

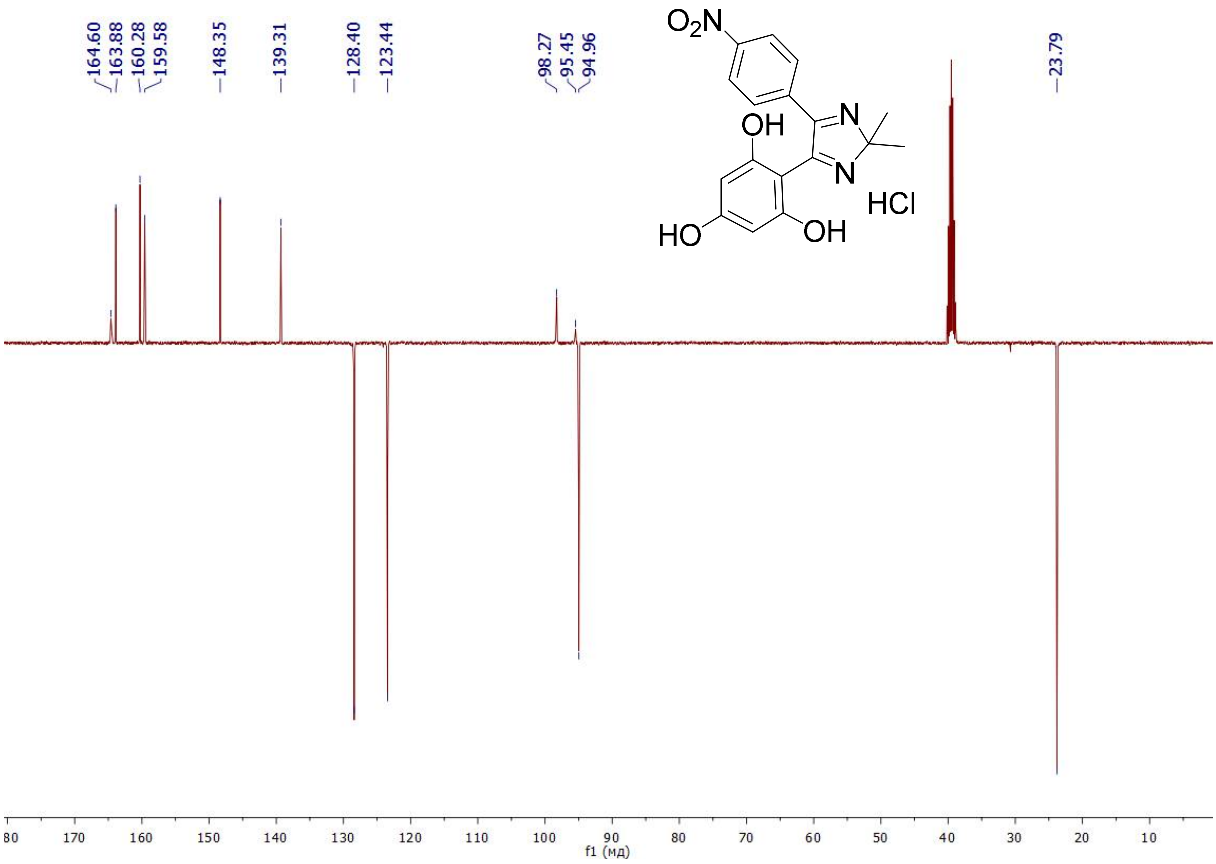

Figure S11. $^{13}C\{^{1}H\}$ NMR spectrum (101 MHz, DMSO-d_6) of compound **Im5Phl**

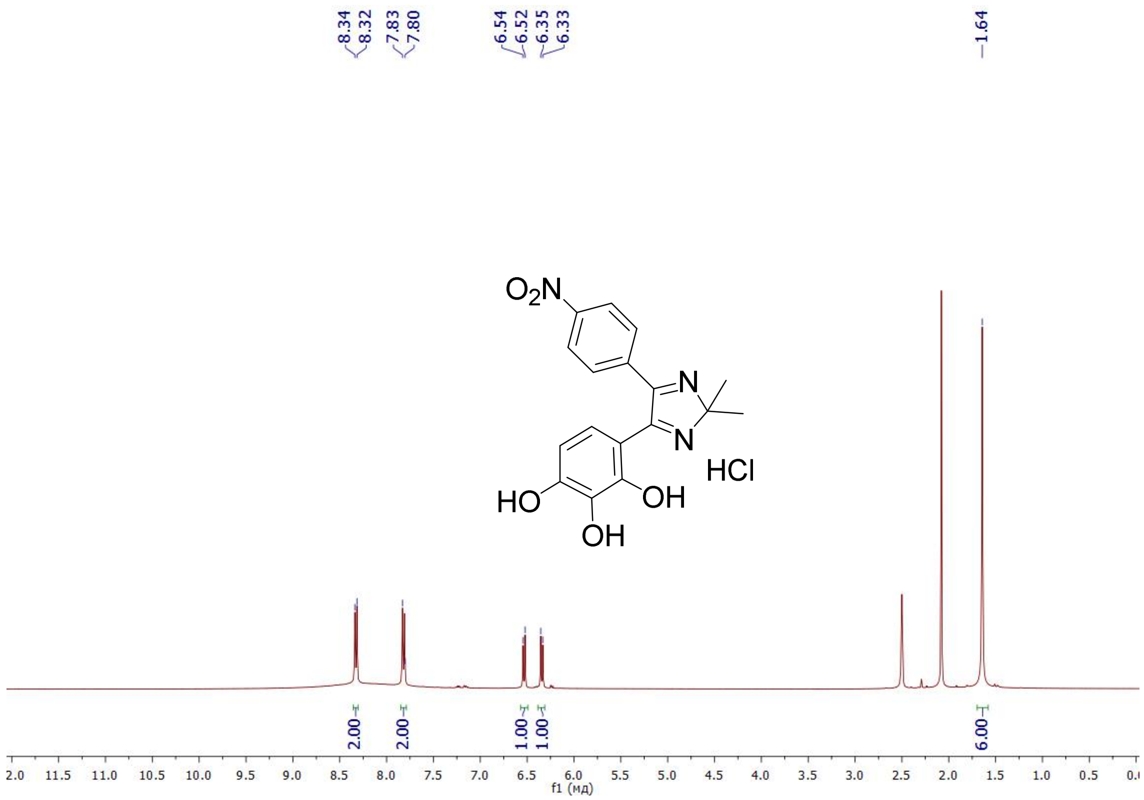

Figure S12. ^{1}H NMR spectrum (400 MHz, DMSO-d$_6$) of compound **Im5Pyr**

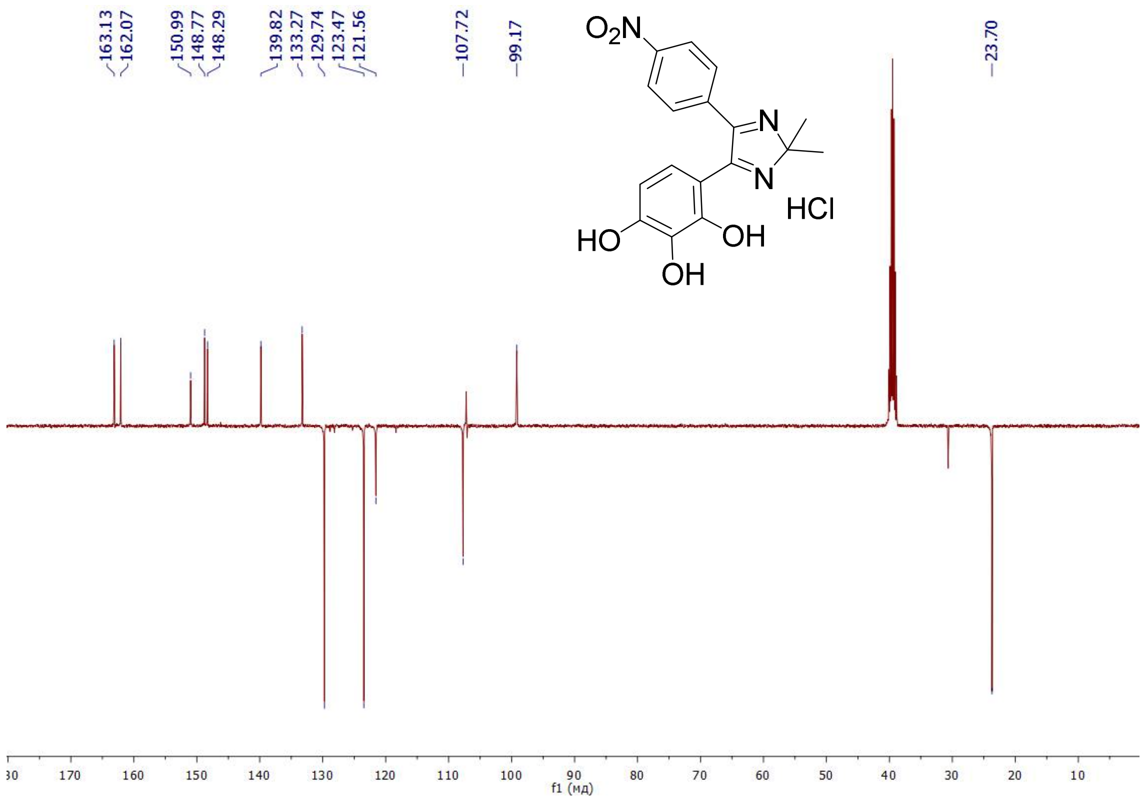

Figure S13. $^{13}C\{^1H\}$ NMR spectrum (101 MHz, DMSO-d$_6$) of compound **Im5Pyr**

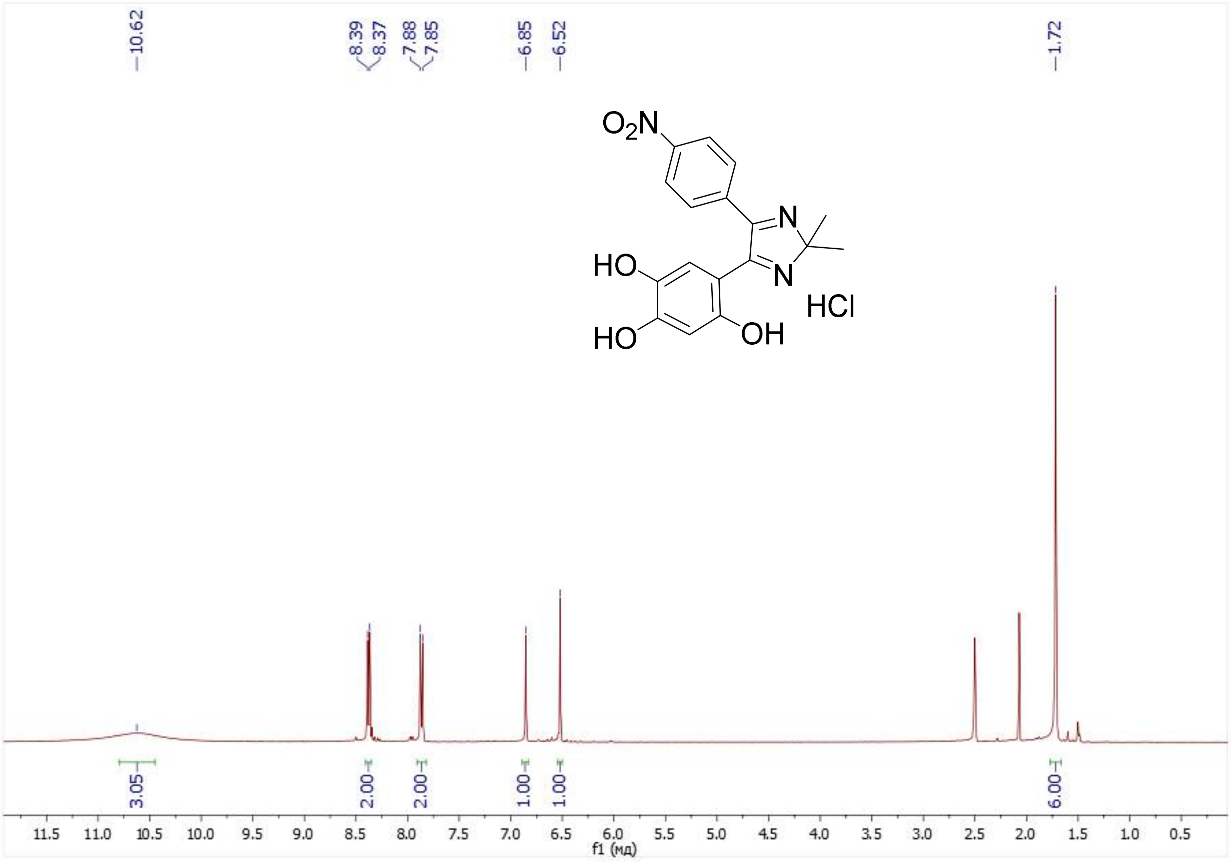

Figure S14. ^{1}H NMR spectrum (400 MHz, DMSO-d6) of compound **Im5Hyd**

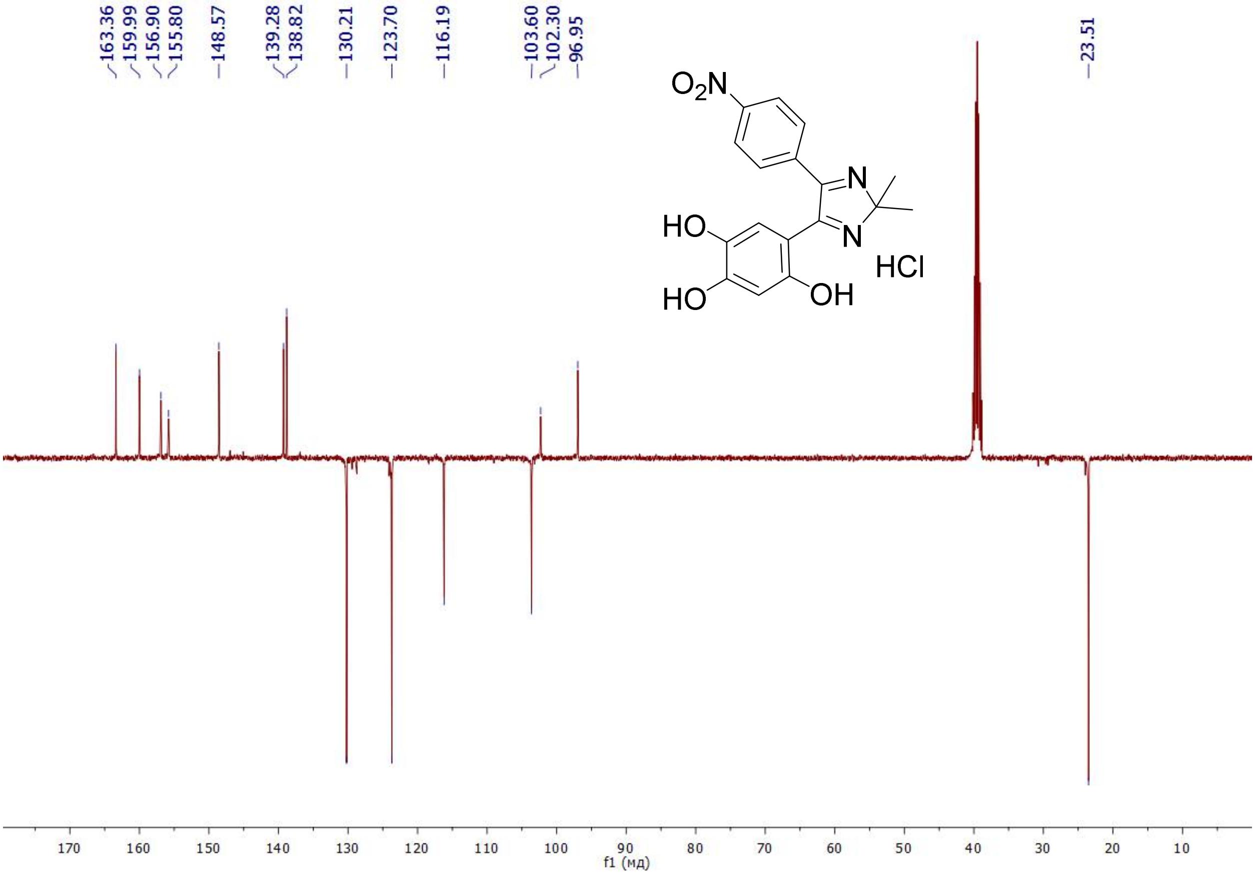

Figure S15. ^{13}C{^{1}H} NMR spectrum (101 MHz, DMSO-d6) of compound **Im5Hyd**

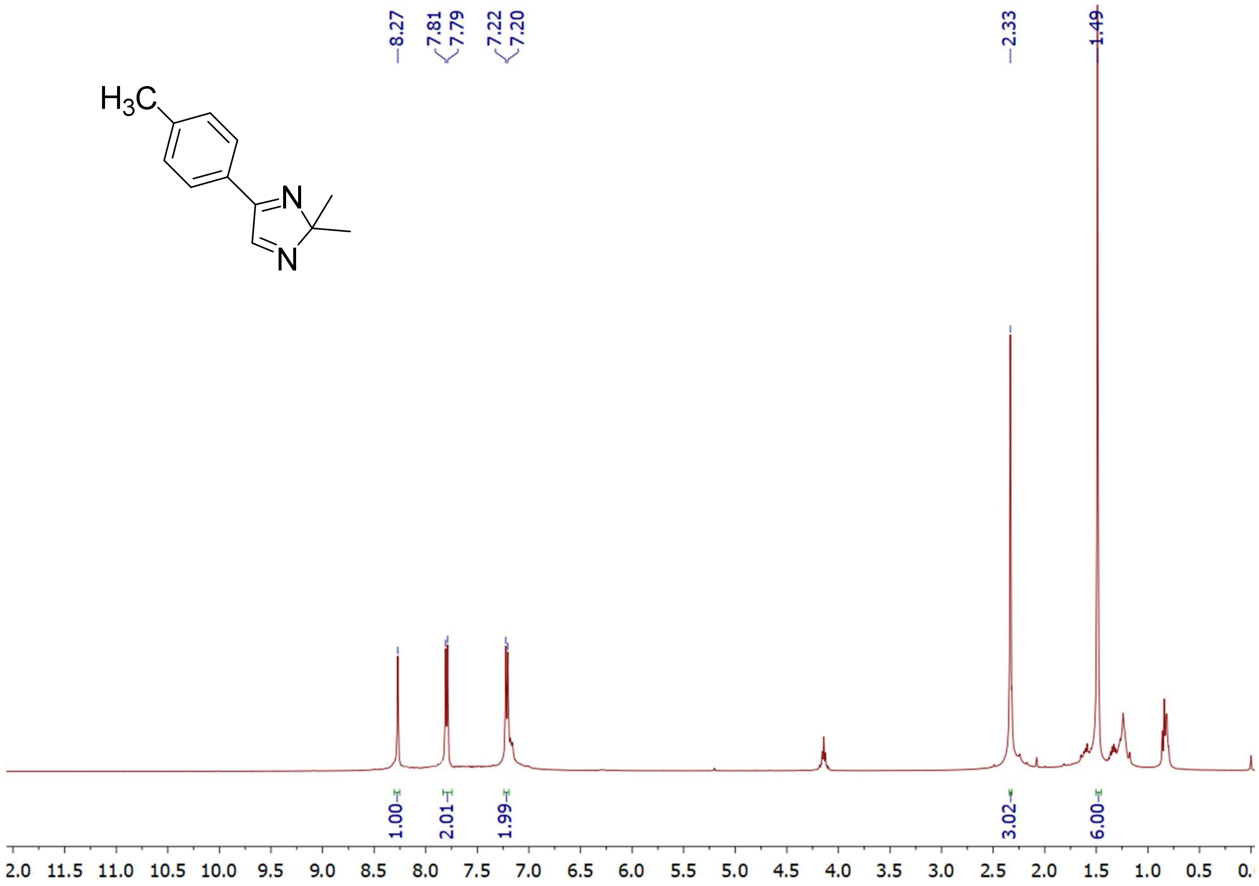

Figure S16. ^{1}H NMR spectrum (400 MHz, $DCCl_3$) of compound **Im1**

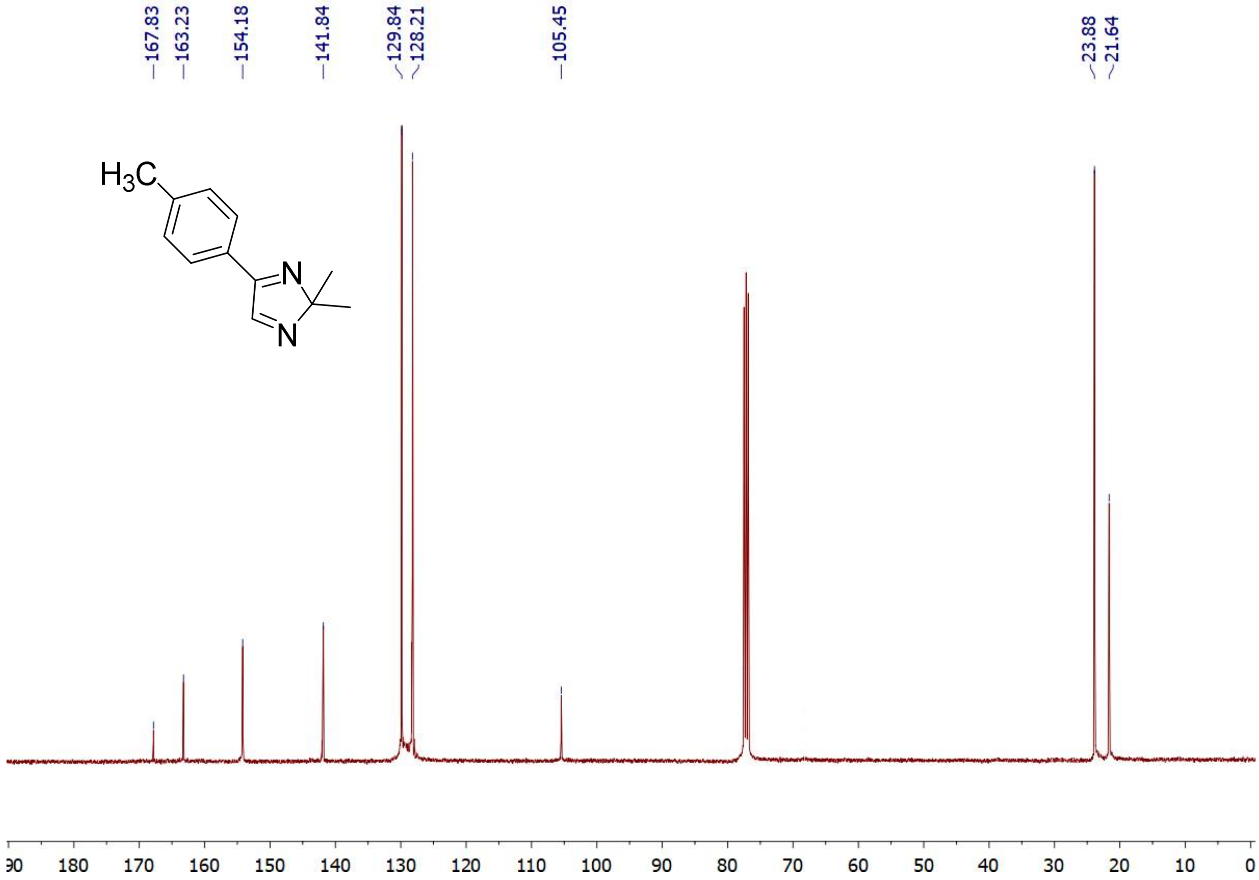

Figure S17. $^{13}C\{^{1}H\}$ NMR spectrum (101 MHz, $DCCl_3$) of compound **Im1**

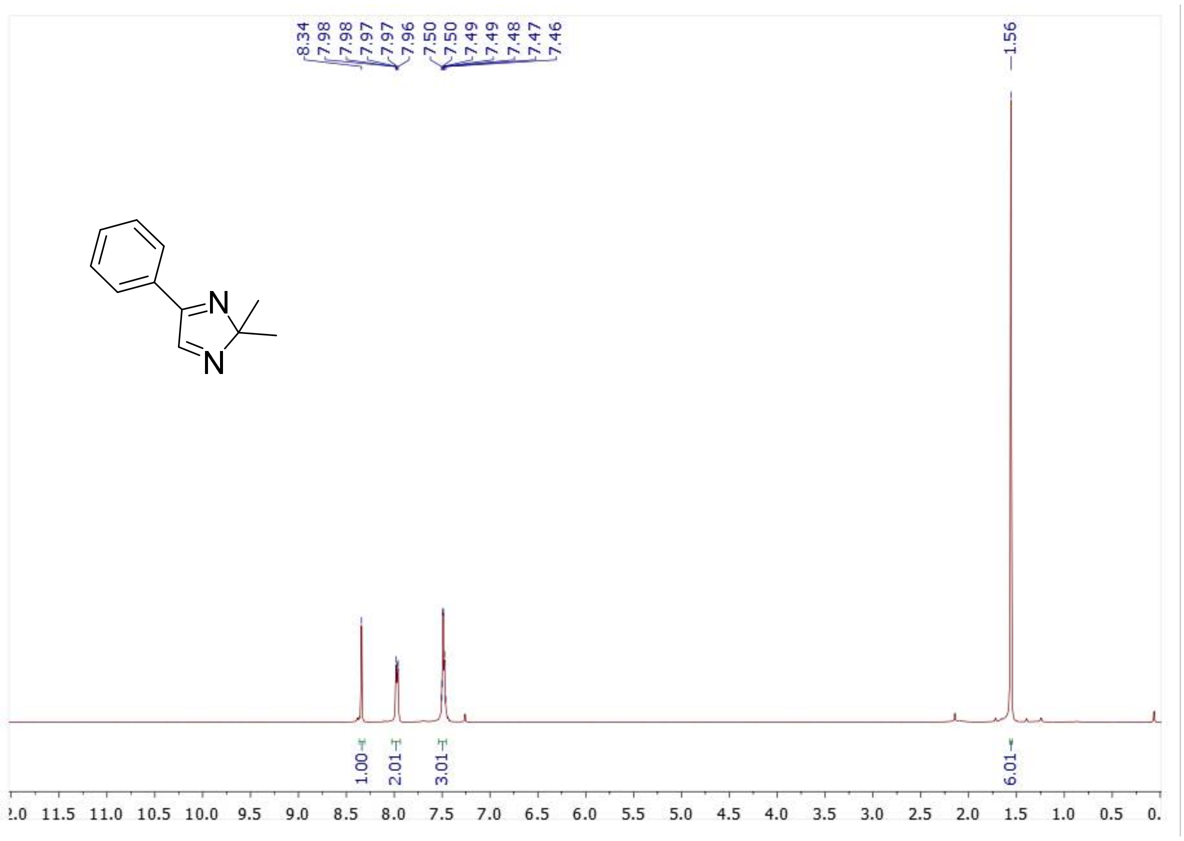

Figure S18. ^{1}H NMR spectrum (400 MHz, $DCCl_3$) of compound **Im2**

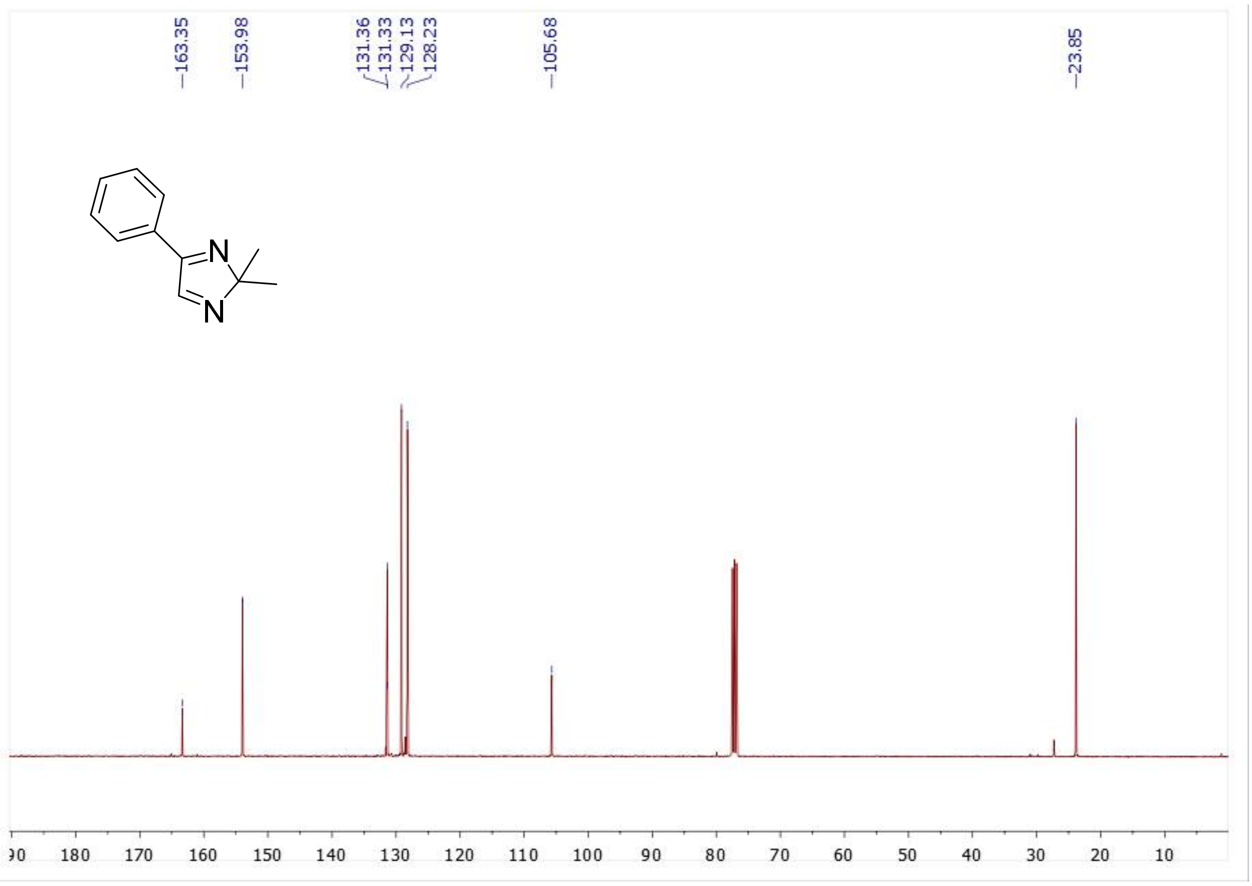

Figure S19. $^{13}C\{^{1}H\}$ NMR spectrum (101 MHz, $DCCl_3$) of compound **Im2**

2. **Correlation between the position of the oxidation peak and AOC for pyrogallol and hydroxyquinol derivatives**

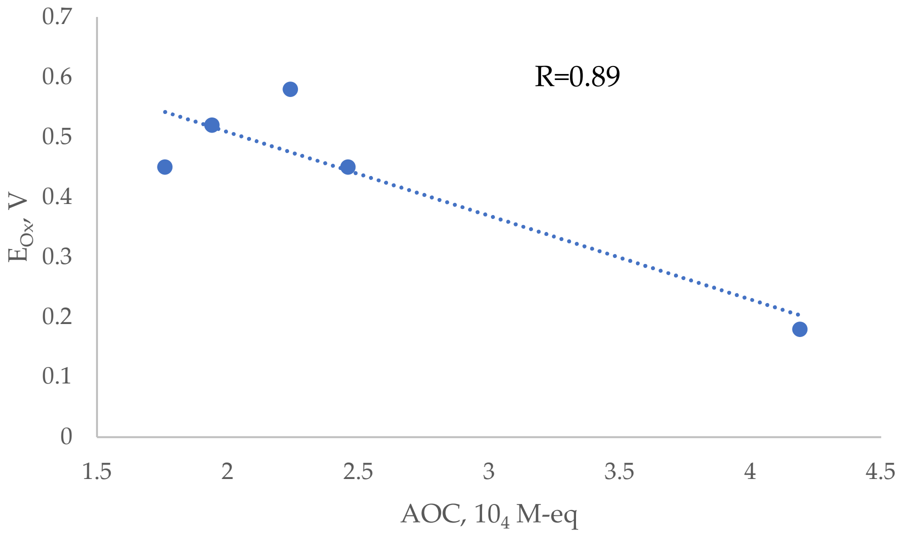

Figure S20. Correlation between the position of the oxidation peak and AOC for imidazolylpyrogallols

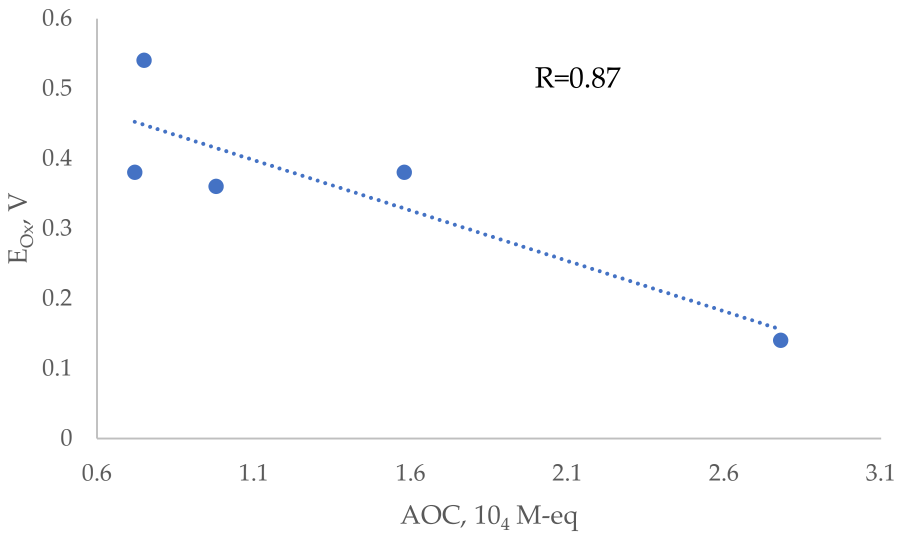

Figure S21. Correlation between the position of the oxidation peak and AOC for imidazolylhydroxyquinols

3. **Antioxidant capacity assay**

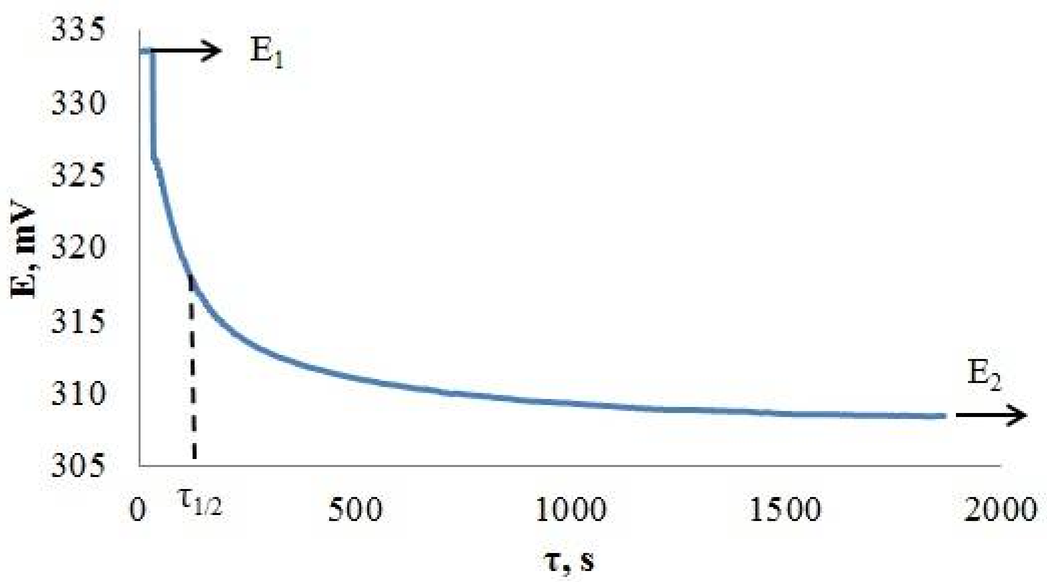

Figure S22. The curve of potential on time with the introduction of 0.1 mM **Im3Phl** into the solution containing $K_3[Fe(CN)_6]/K_4[Fe(CN)_6]$ in a ratio of 0.01 M / 0.1 mM, respectively (pH = 7.4 PBS)

4. **Antiradical capacity assay**

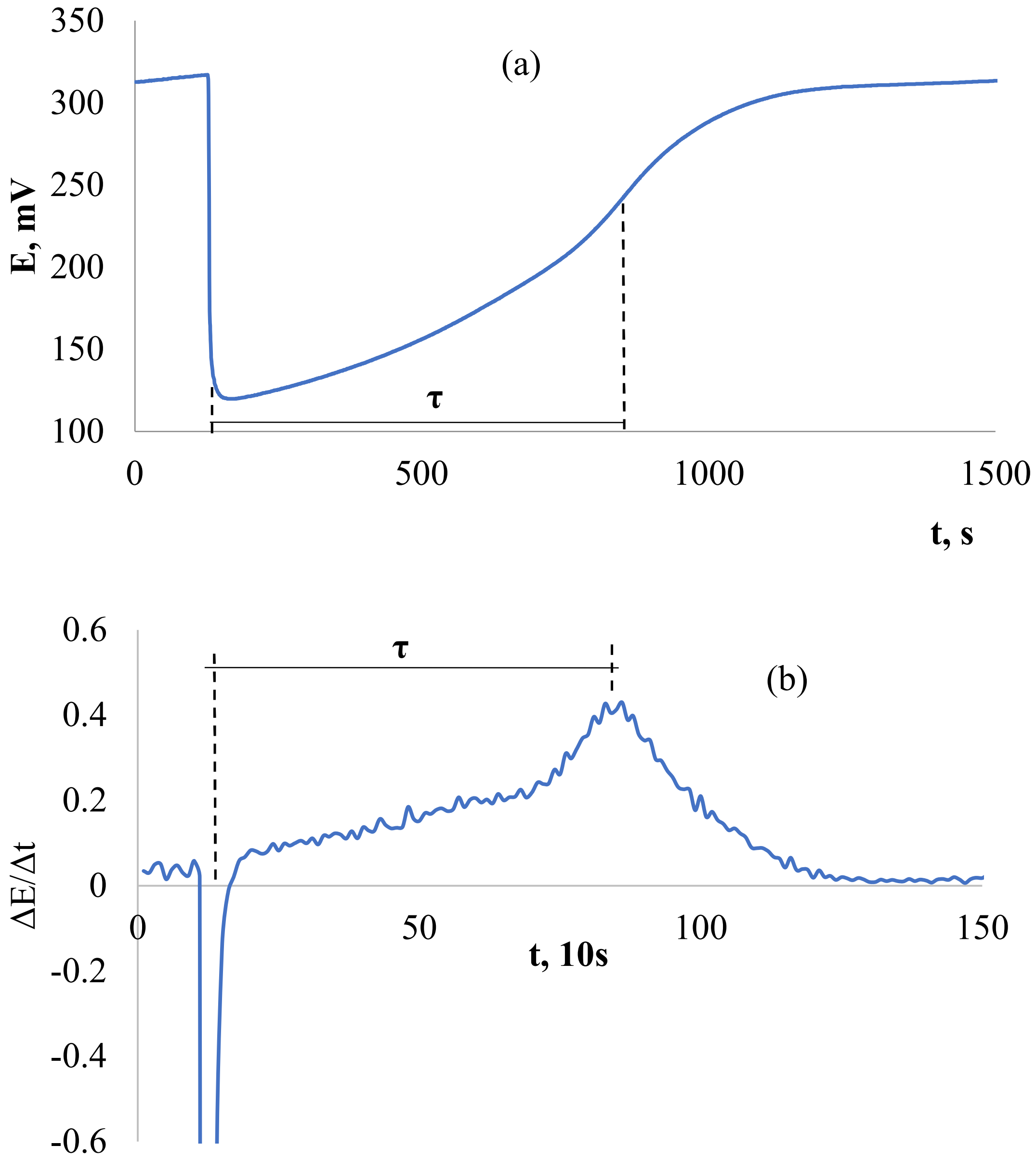

Figure S23. Integral (a) and differential (b) kinetic curves of potential on time when adding 0.1 mM **Im3Phl** to 0.1 M AAPH (pH = 7.4 PBS)
